# Supplementary material for: An Integrative Glycomic Approach for Quantitative Meat Species Profiling
Source: Foods. 2022 Jun 30;11(13):1952. doi: 10.3390/foods11131952 (PMC9265272; doi:10.3390/foods11131952)
Supplement: Supplementary file 1 [file foods-11-01952-s001.zip › foods-1754860-supplementary.pdf]

Supplementary Figures

Representative of N-glycan MS spectra

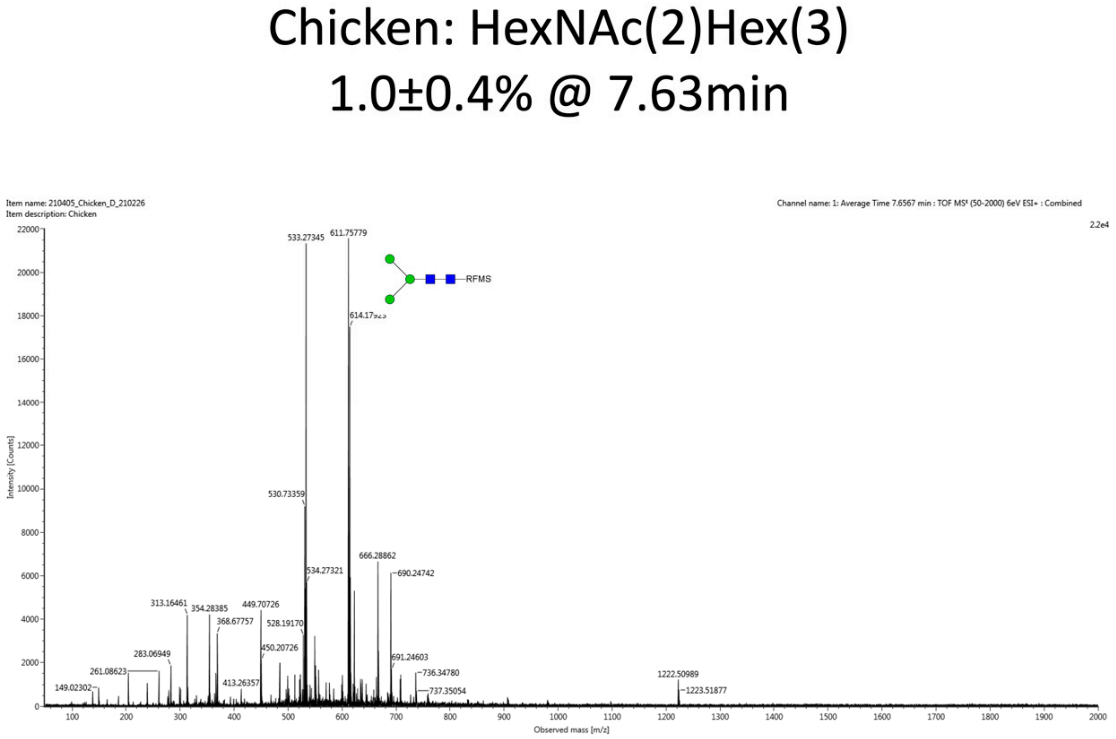

Figure S1. Chicken: HexNAc(2)Hex(3) 1.0 ± 0.4%, 7.63 min.

Chicken: HexNAc(2)Hex(4)
2.9±0.8% @ 9.93min

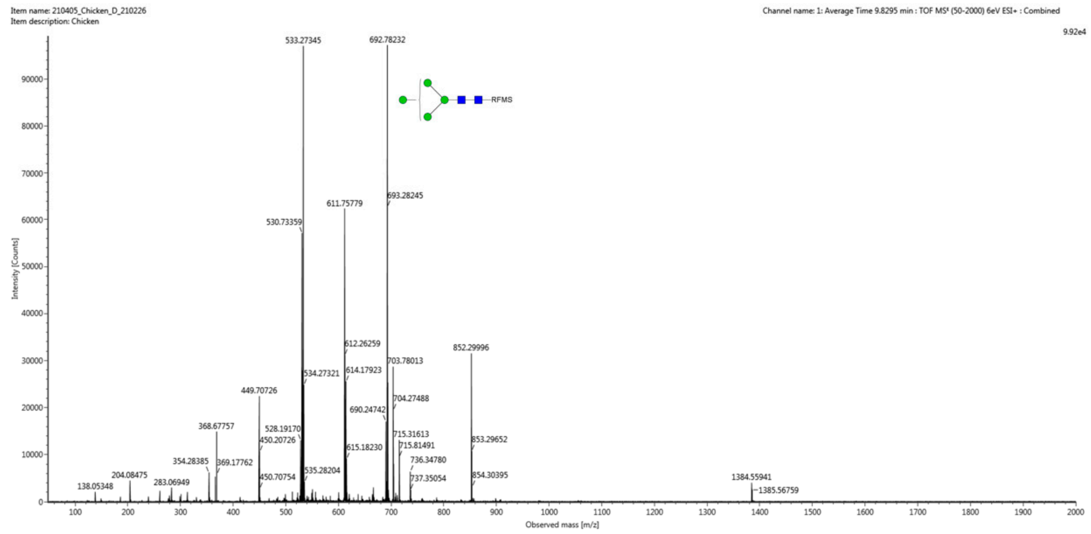

Figure S2. Chicken: HexNAc(2)Hex(4) 2.9 ± 0.8%, 9.93 min.

Chicken: HexNAc(4)Hex(3)Fuc(1)
1.1±0.2% @ 11.48min

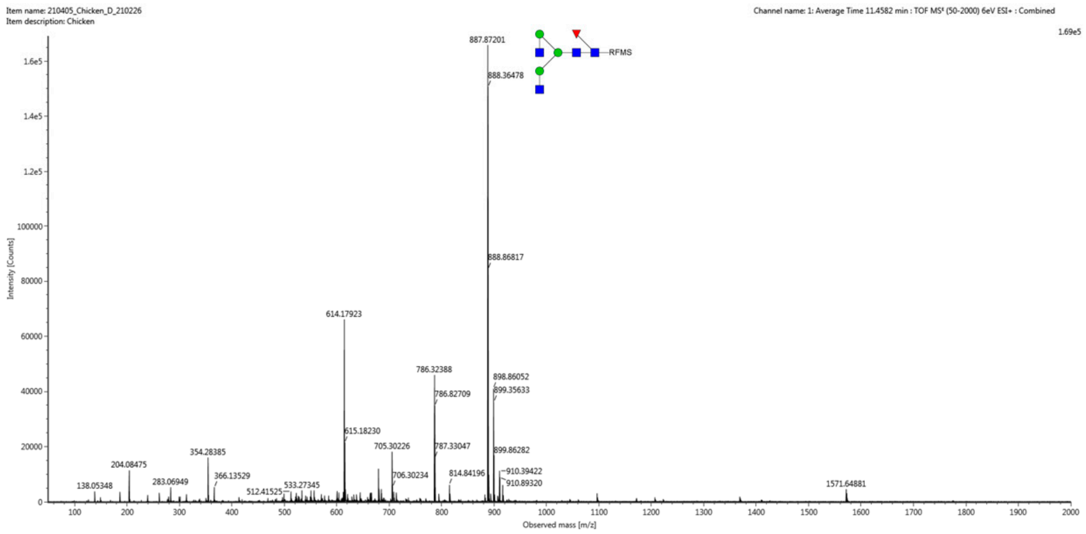

Figure S3. Chicken: HexNAc(4)Hex(3)Fuc(1) 1.1 ± 0.2%, 11.48 min.

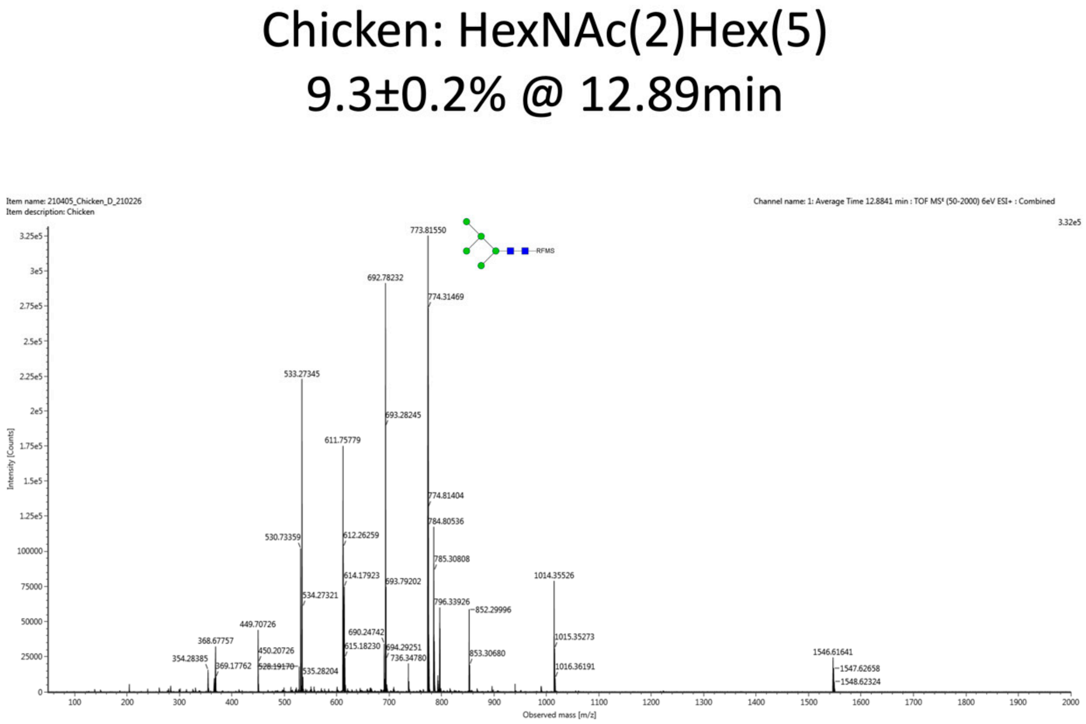

Figure S4. Chicken: HexNAc(2)Hex(5) 9.3 ± 0.2%, 12.89 min.

Chicken: HexNAc(5)Hex(4)Fuc(1)
1.6±0.1% @ 14.47min

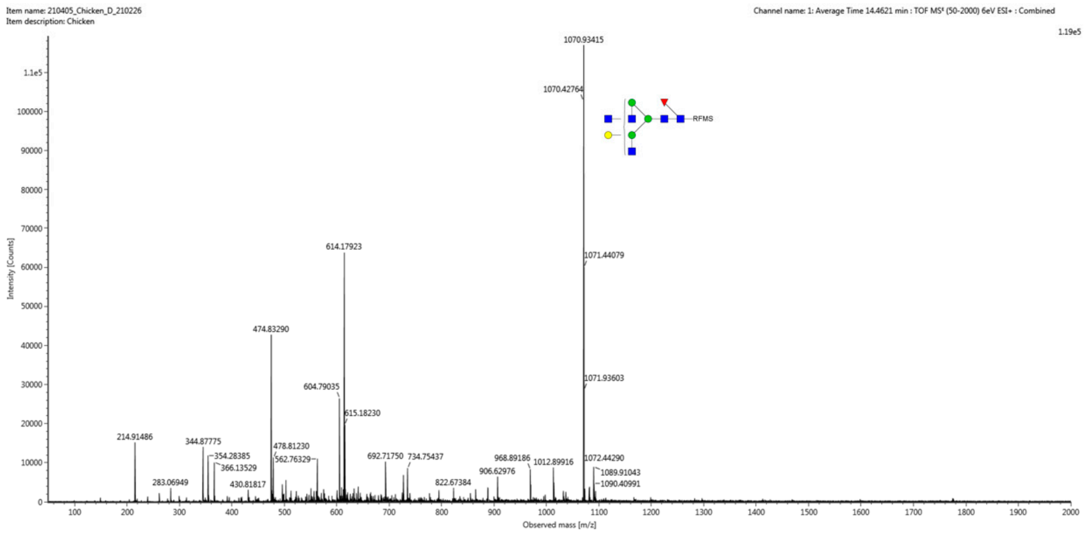

Figure S5. Chicken: HexNAc(5)Hex(4)Fuc(1) 1.6 ± 0.1%, 14.47 min.

Chicken: HexNAc(4)Hex(5)
2.0±0.2% @ 15.57min

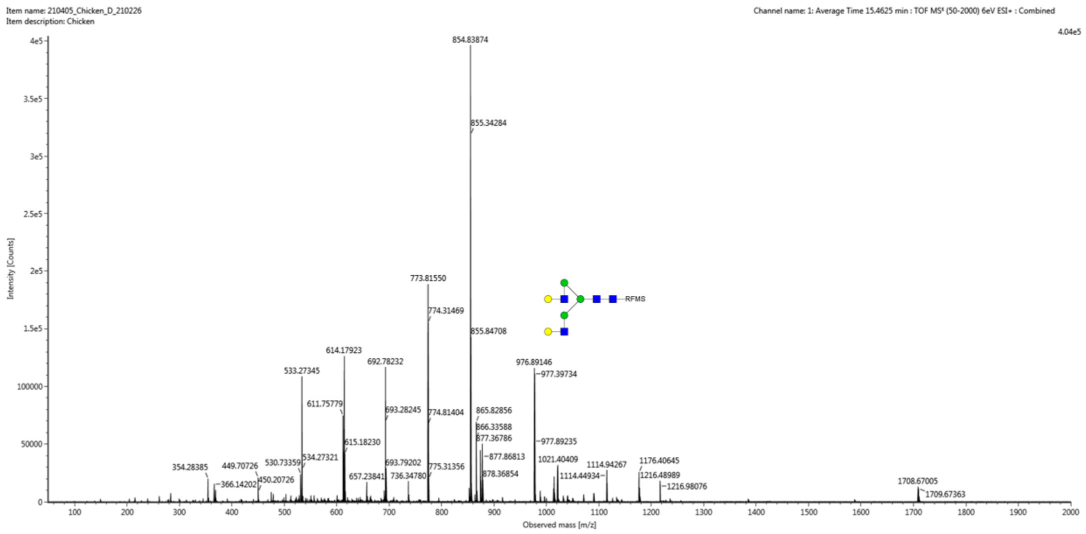

Figure S6. Chicken: HexNAc(4)Hex(5) 2.0 ± 0.2%, 15.57 min.

Chicken: HexNAc(2)Hex(6)
8.1±0.8% @ 15.57min

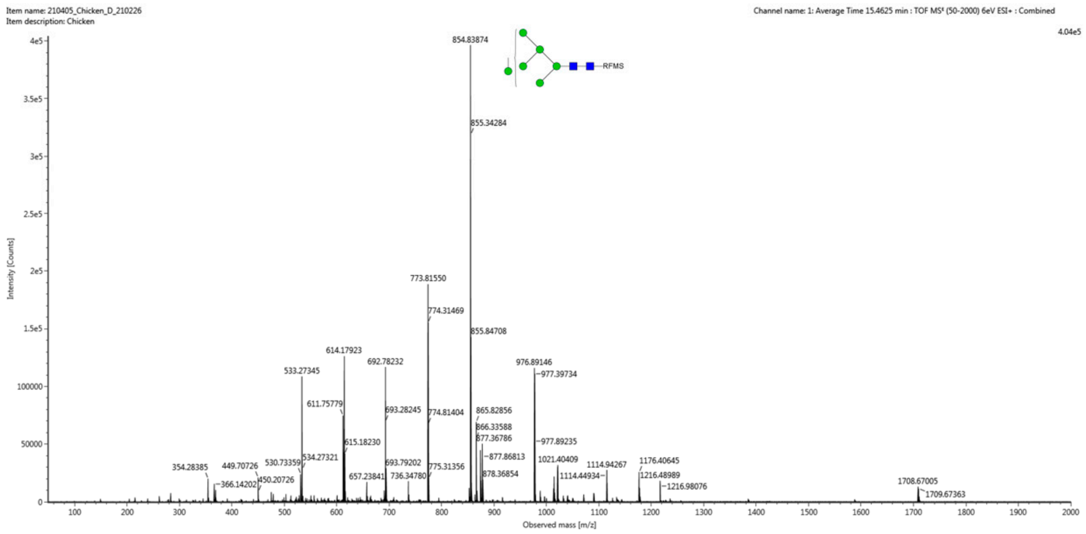

Figure S7. Chicken: HexNAc(2)Hex(6) 8.1 ± 0.8%, 15.57 min.

Chicken: HexNAc(4)Hex(5)Fuc(1)
2.2±0.4% @ 16.29min

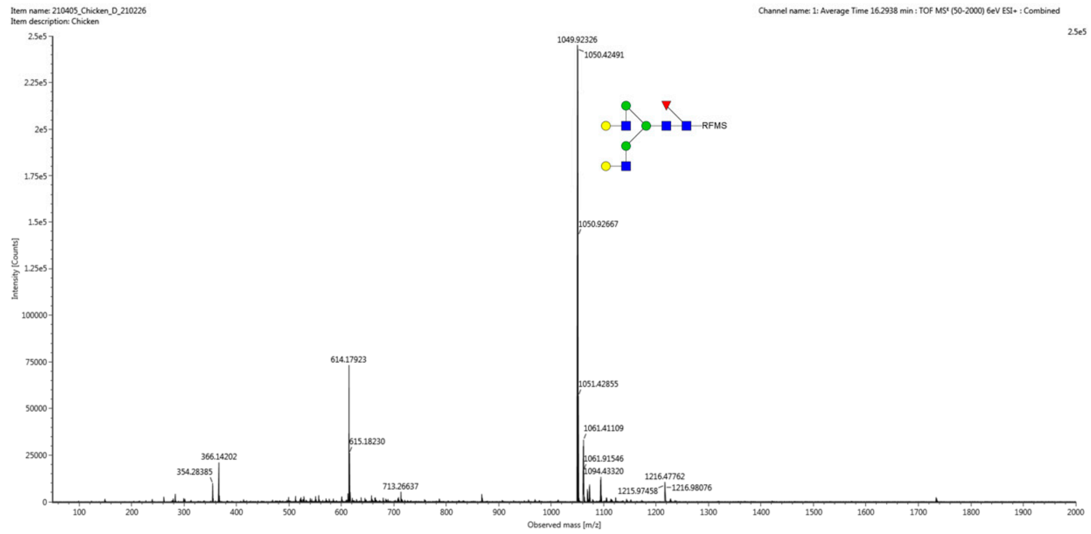

Figure S8. Chicken: HexNAc(4)Hex(5)Fuc(1) 2.2 ± 0.4%, 16.29 min.

Chicken: HexNAc(5)Hex(5)Fuc(1)
5.9±0.5% @ 16.74min

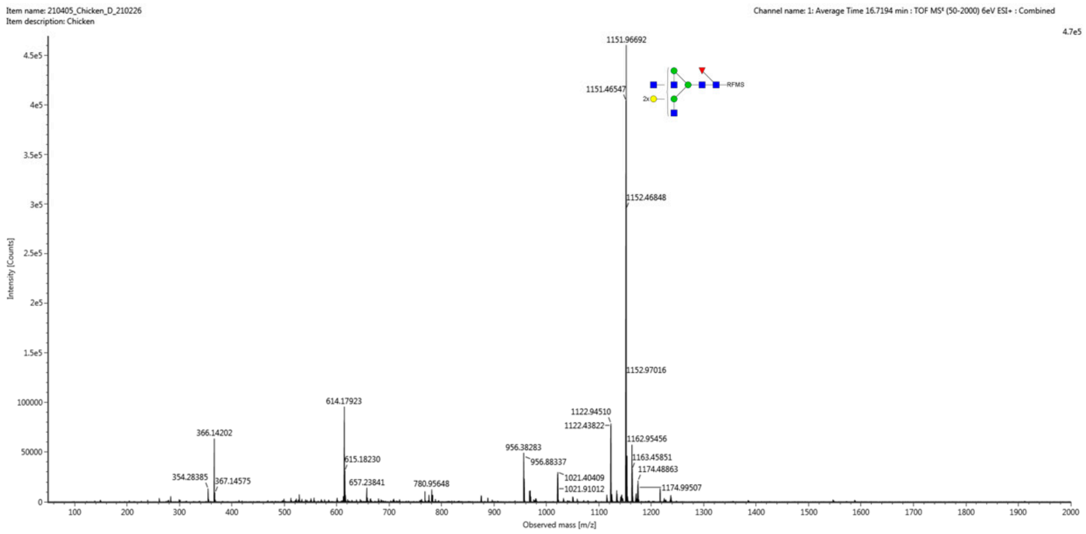

Figure S9. Chicken: HexNAc(5)Hex(5)Fuc(1) 5.9 ± 0.5%, 16.74 min.

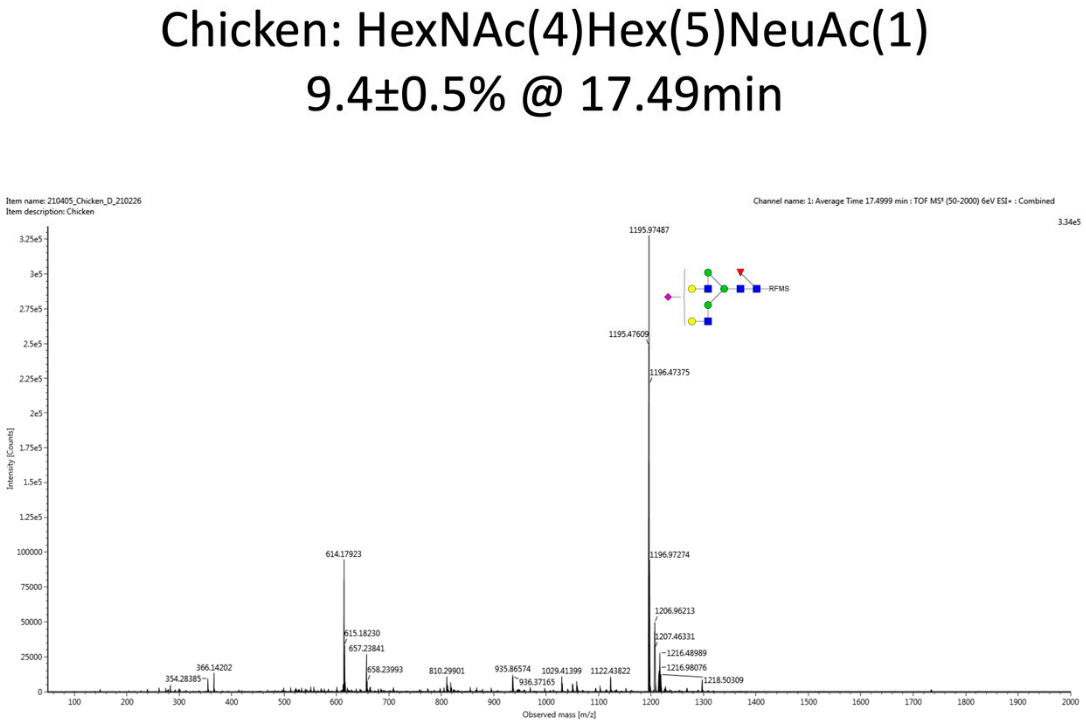

Figure S10. Chicken: HexNAc(4)Hex(5)Fuc(1) 9.4 ± 0.5%, 17.49 min.

Chicken: HexNAc(4)Hex(5)Fuc(1)NeuAc(1)
6.2±0.6% @ 17.98min

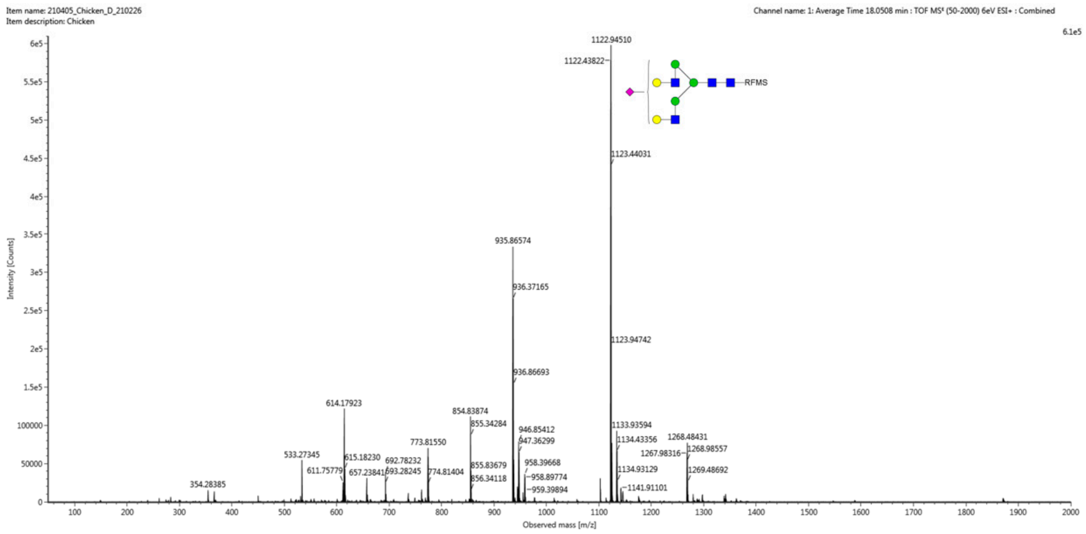

Figure S11. Chicken: HexNAc(4)Hex(5)Fuc(1)NeuAc(1) 6.2 ± 0.6%, 17.98 min.

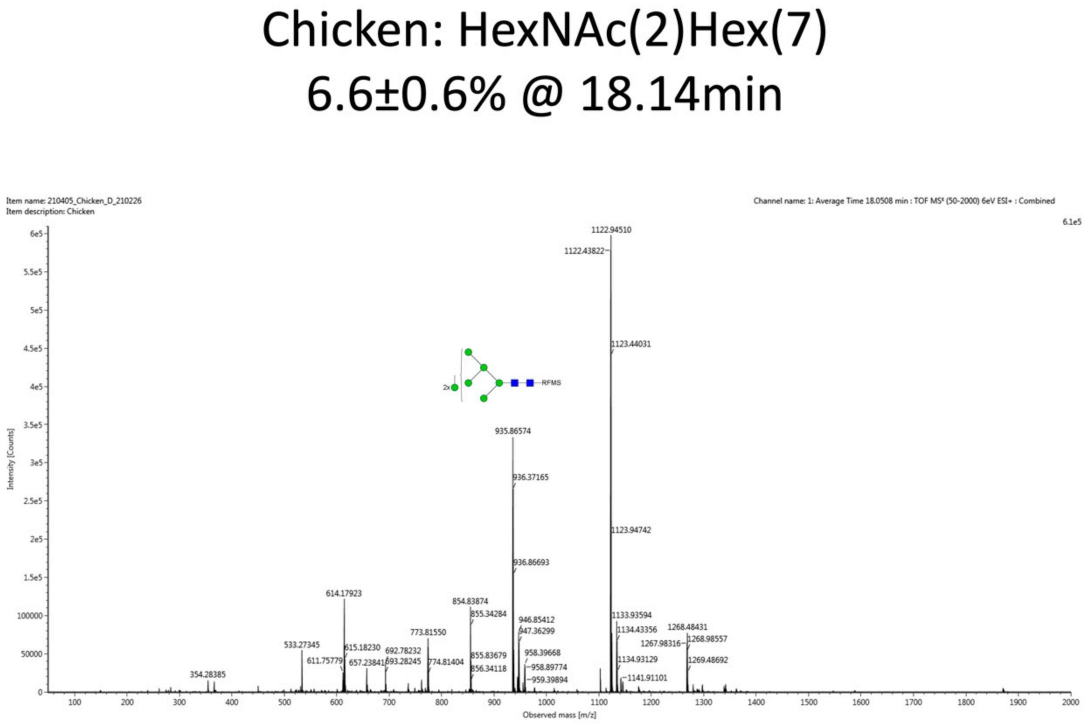

Figure S12. Chicken: HexNAc(2)Hex(7) 6.6 ± 0.6%, 18.14 min.

Chicken: HexNAc(4)Hex(5)Fuc(1)NeuAc(2)
4.3±0.5% @ 18.67min

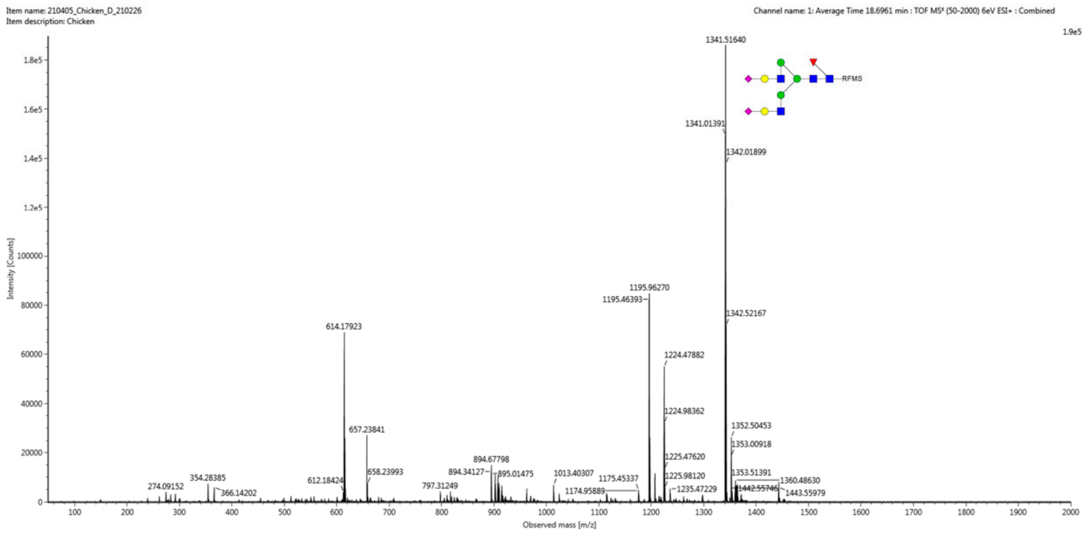

Figure S13. Chicken: HexNAc(4)Hex(5)Fuc(1)NeuAc(2) 4.3 ± 0.5%, 18.67 min.

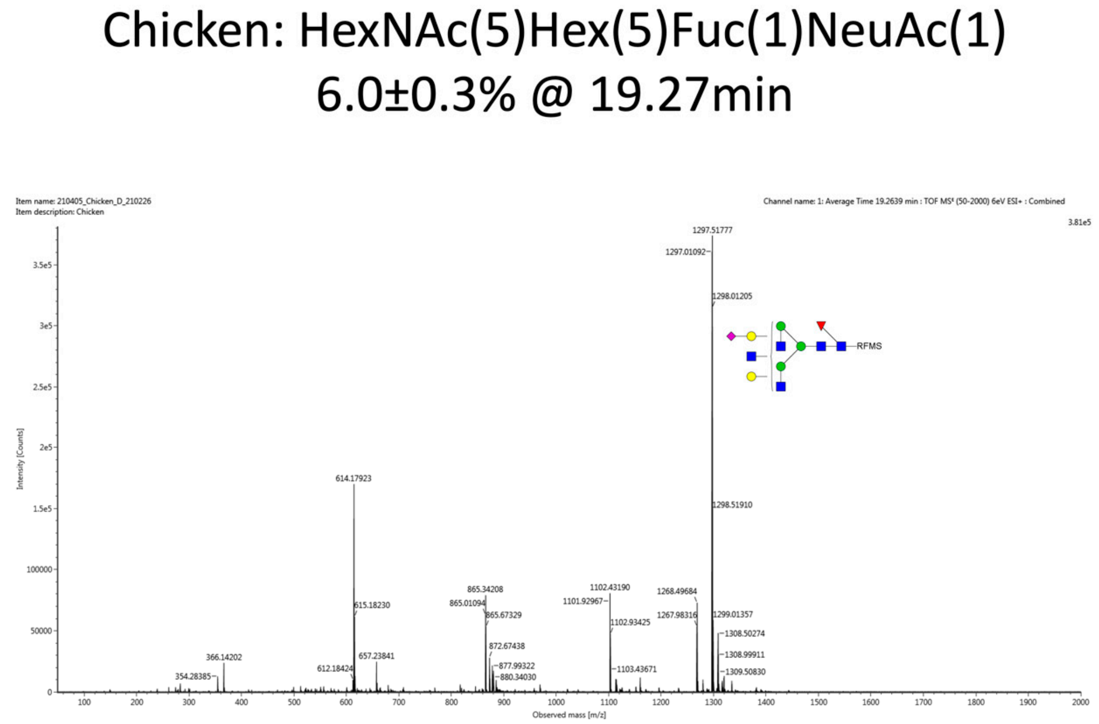

Figure S14. Chicken: HexNAc(5)Hex(5)Fuc(1)NeuAc(1) 6.0 ± 0.3%, 19.27 min.

Chicken: HexNAc(4)Hex(5)NeuAc(2)
11.9±1.0% @ 20.2min

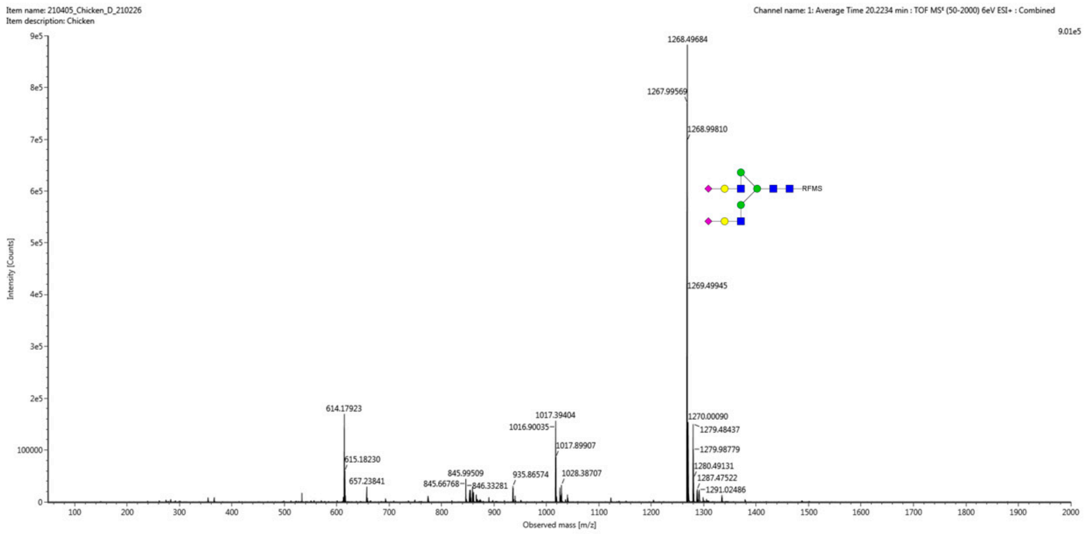

Figure S15. Chicken: HexNAc(4)Hex(5)NeuAc(2) 11.9 ± 1.0%, 20.2 min.

Chicken: HexNAc(2)Hex(8)
10.8±1.1% @ 20.51min

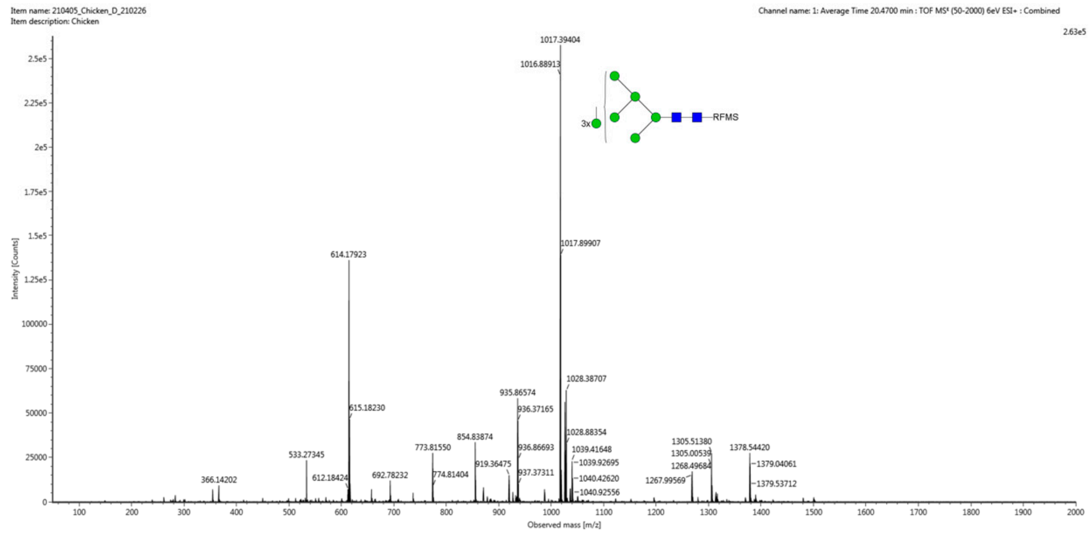

Figure S16. Chicken: HexNAc(2)Hex(8) 10.8 ± 1.1%, 20.51 min.

Chicken: HexNAc(2)Hex(9)
9.2±0.7% @ 22.31min

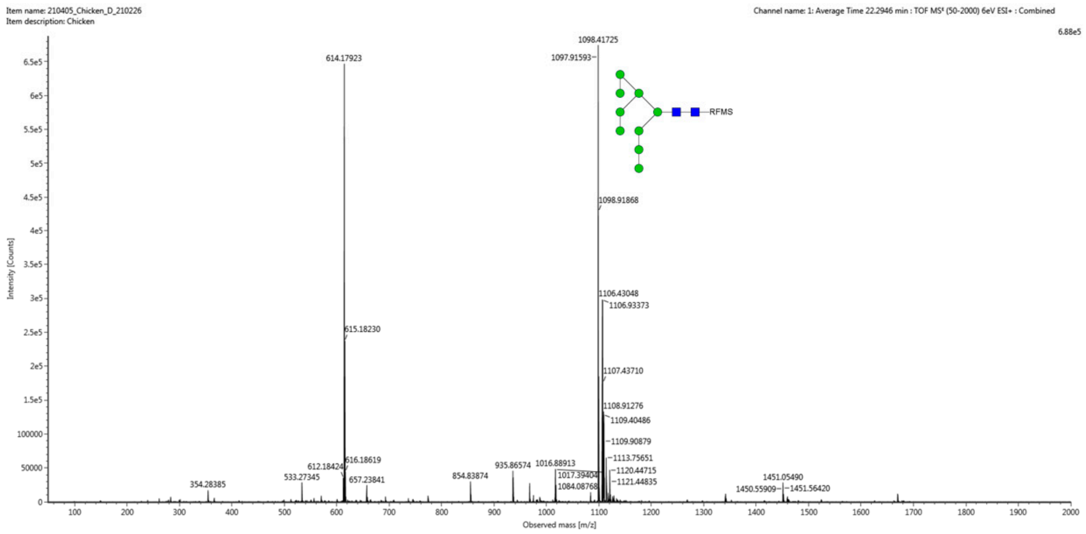

Figure S17. Chicken: HexNAc(2)Hex(9) 9.2 ± 0.7%, 22.31 min.

Pork: HexNAc(2)Hex(3)
0.3±0.3% @ 7.6min

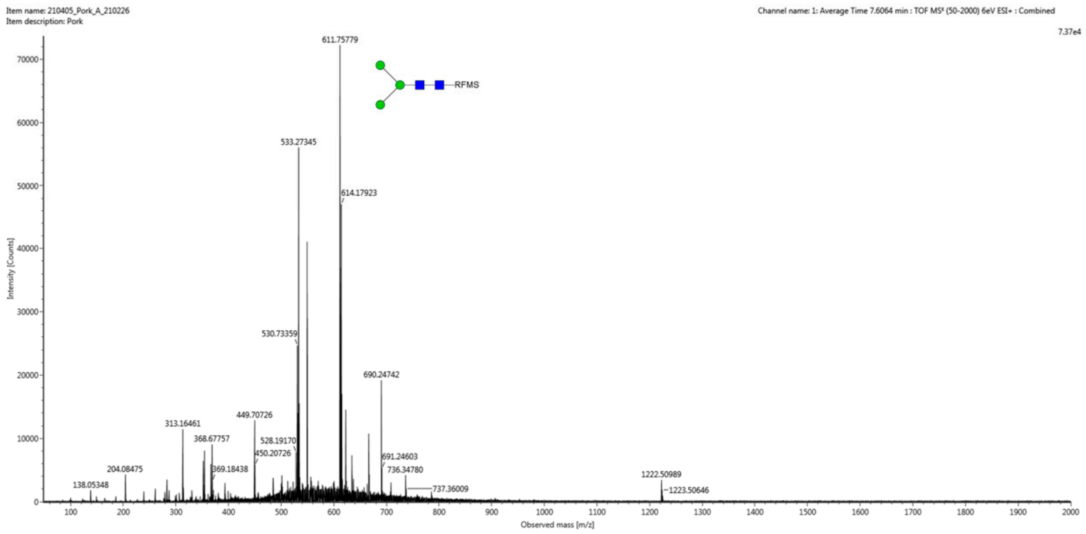

Figure S18. Pork: HexNAc(2)Hex(3) 0.3 ± 0.3%, 7.6 min.

Pork: HexNAc(2)Hex(4)
0.5±0.5% @ 9.91min

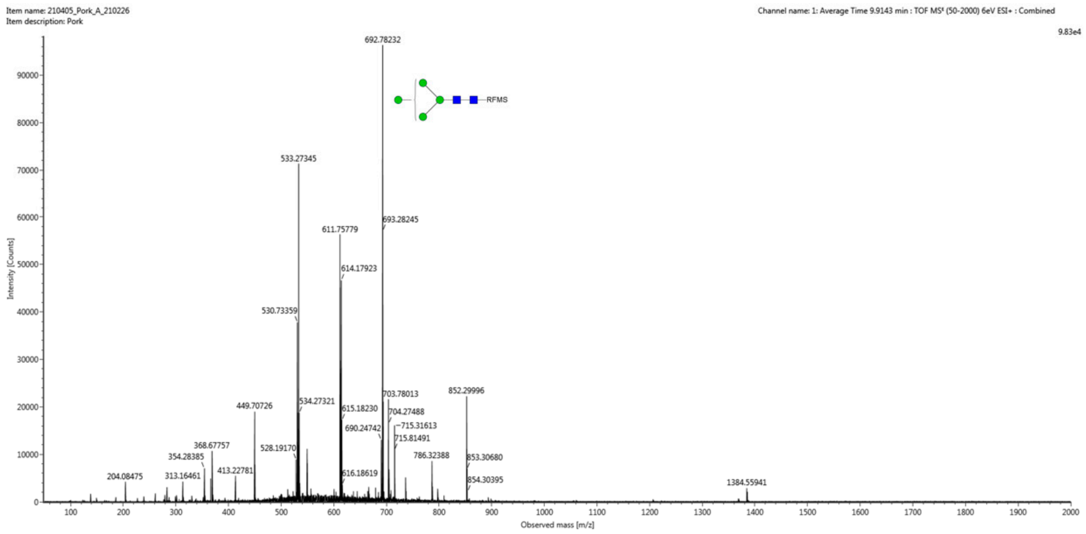

Figure S19. Pork: HexNAc(2)Hex(4) 0.5 ± 0.5%, 9.91 min.

Pork: HexNAc(4)Hex(3)Fuc(1)
6.3±0.2% @ 11.46min

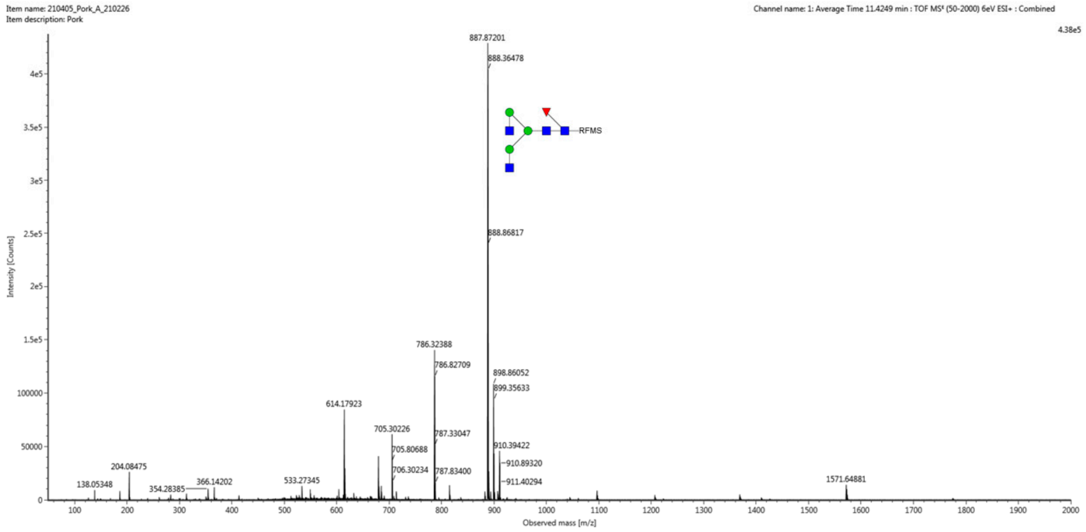

Figure S20. Pork: HexNAc(4)Hex(3)Fuc(1) 6.3 ± 0.2%, 11.46 min.

Pork: HexNAc(2)Hex(5)
4.3±0.5% @ 12.87min

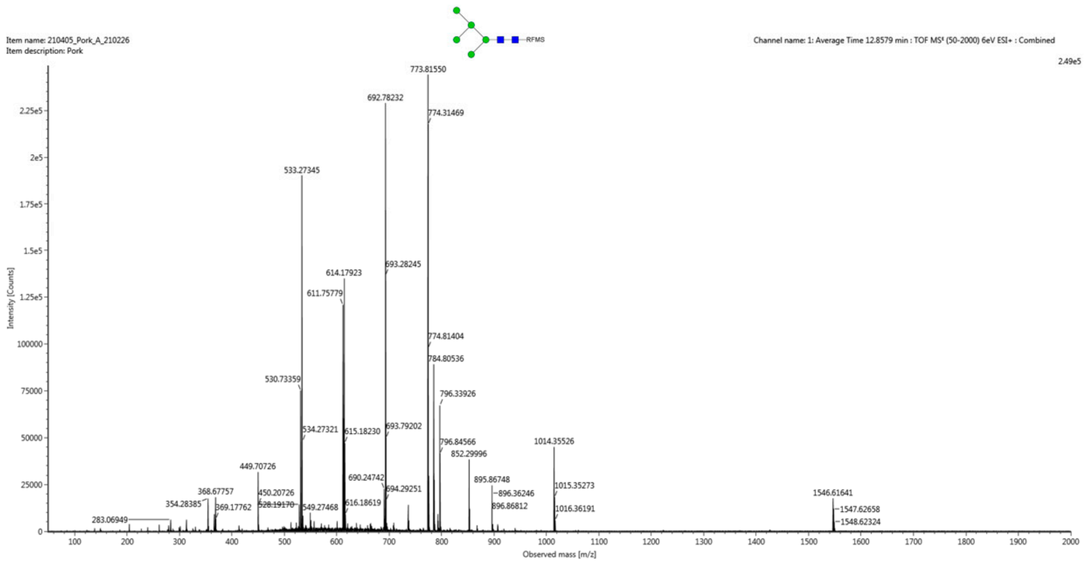

Figure S21. Pork: HexNAc(2)Hex(5)Fuc 4.3 ± 0.5%, 12.87 min.

Pork: HexNAc(4)Hex(4)Fuc(1)
1.2±0.4% @ 13.7min 14.07min

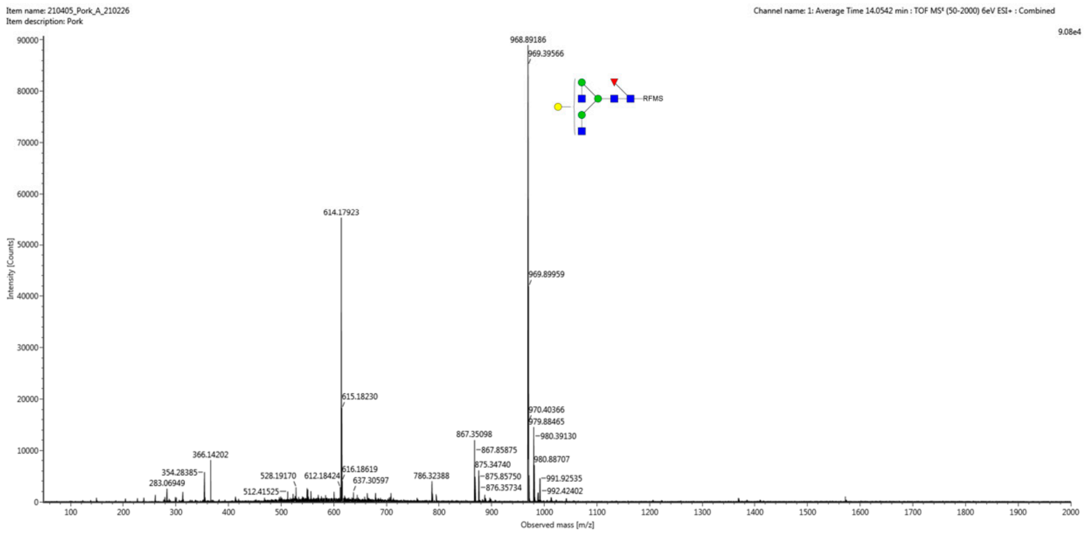

Figure S22. Pork: HexNAc(4)Hex(4)Fuc(1) 1.2 ± 0.4%, 13.7 min, 14.07 min.

Pork: HexNAc(4)Hex(5)
0.3±0.1% @ 15.55min

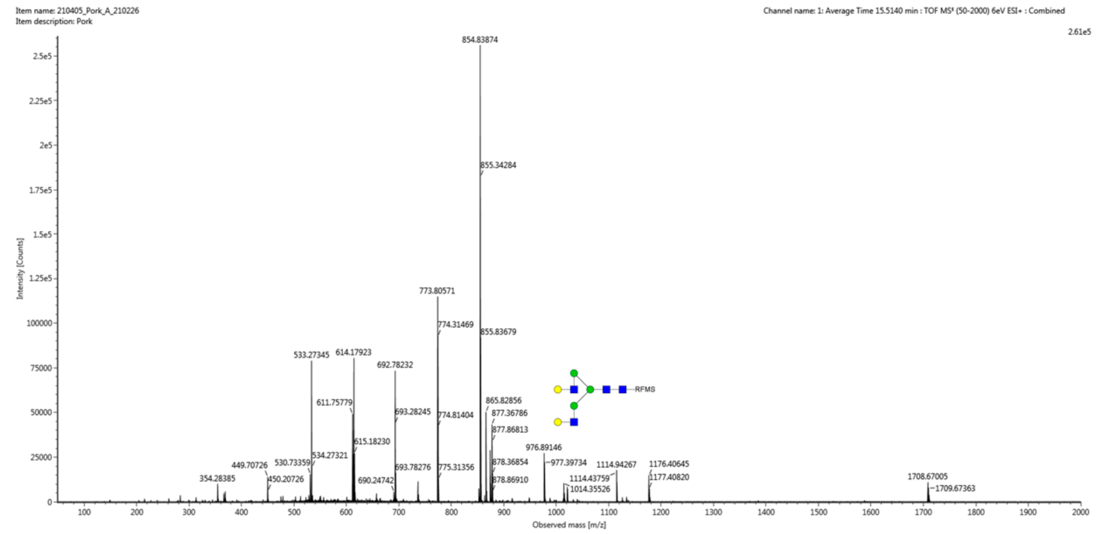

Figure S23. Pork: HexNAc(4)Hex(5) 0.3 ± 0.1%, 15.55 min.

Pork: HexNAc(2)Hex(6)
4.5±1.0% @ 15.55min

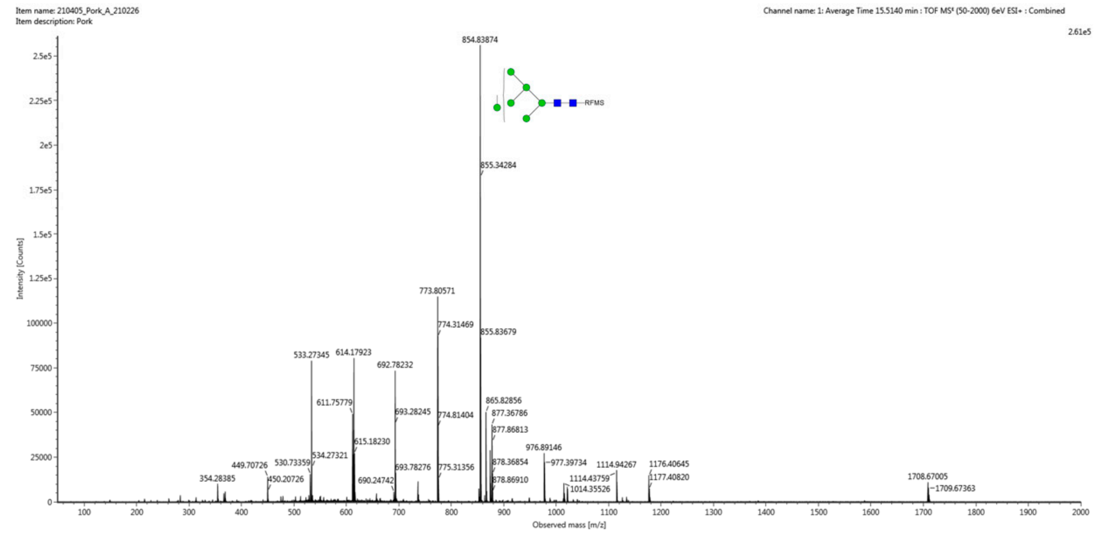

Figure S24. Pork: HexNAc(2)Hex(6) 4.5 ± 1.0%, 15.55 min.

Pork: HexNAc(4)Hex(5)Fuc(1)NeuAc(1)
0.2±0.2% @ 17.46min

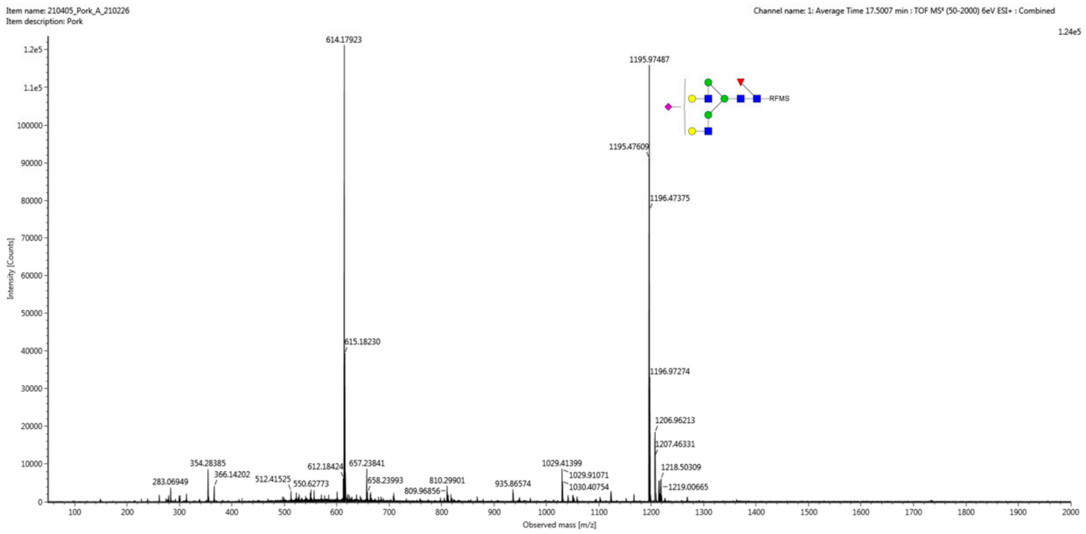

Figure S25. Pork: HexNAc(4)Hex(3)Fuc(1)NeuAc(1) 0.2 ± 0.2%, 17.46 min.

Pork: HexNAc(2)Hex(7)
3.9±0.6% @ 18.07min

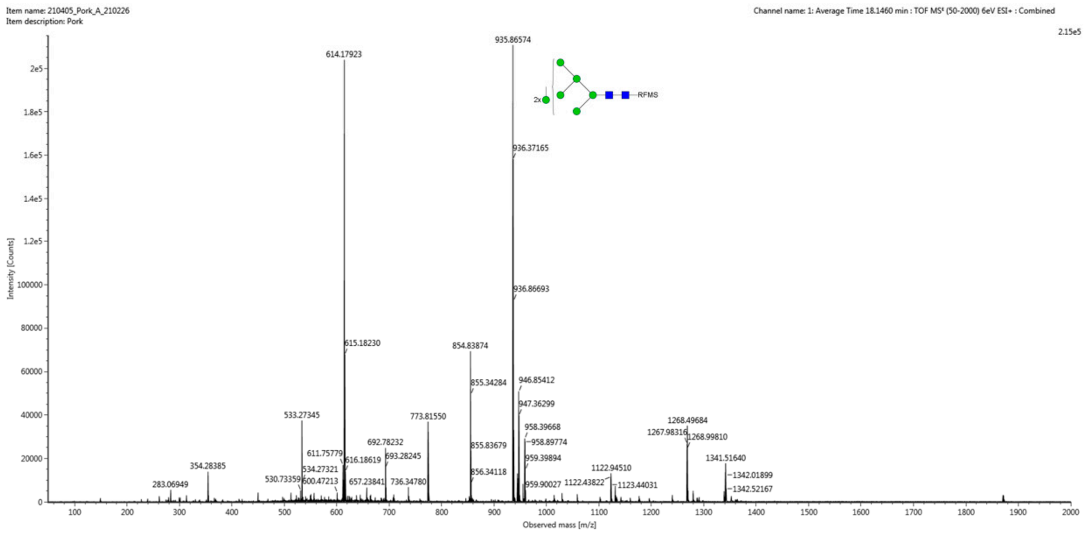

Figure S26. Pork: HexNAc(2)Hex(7) 3.9 ± 0.6%, 18.07 min.

Pork: HexNAc(4)Hex(5)Fuc(1)NeuAc(2)
60.7±6.3% @ 18.64min

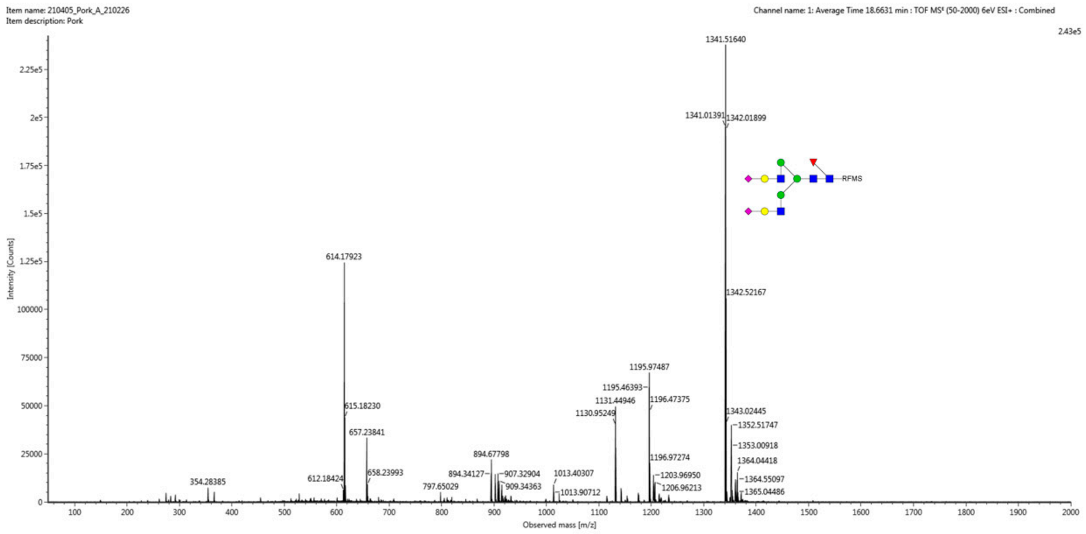

Figure S27. Pork: HexNAc(4)Hex(5)Fuc(1)NeuAc(2) 60.7 ± 6.3%, 18.64 min.

Pork: HexNAc(4)Hex(6)Fuc(1)NeuAc(1)
1.2±0.5% @ 19.47min

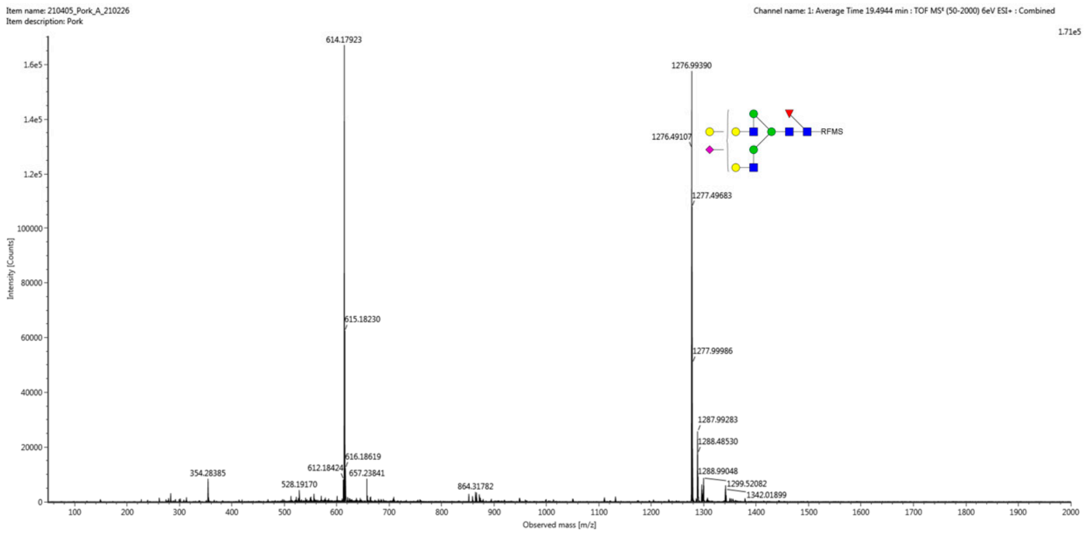

Figure S28. Pork: HexNAc(4)Hex(6)Fuc(1)NeuAc(1) 1.2 ± 0.5%, 19.47 min.

Pork: HexNAc(4)Hex(5)NeuAc(2)
2.3±0.2% @ 20.19min

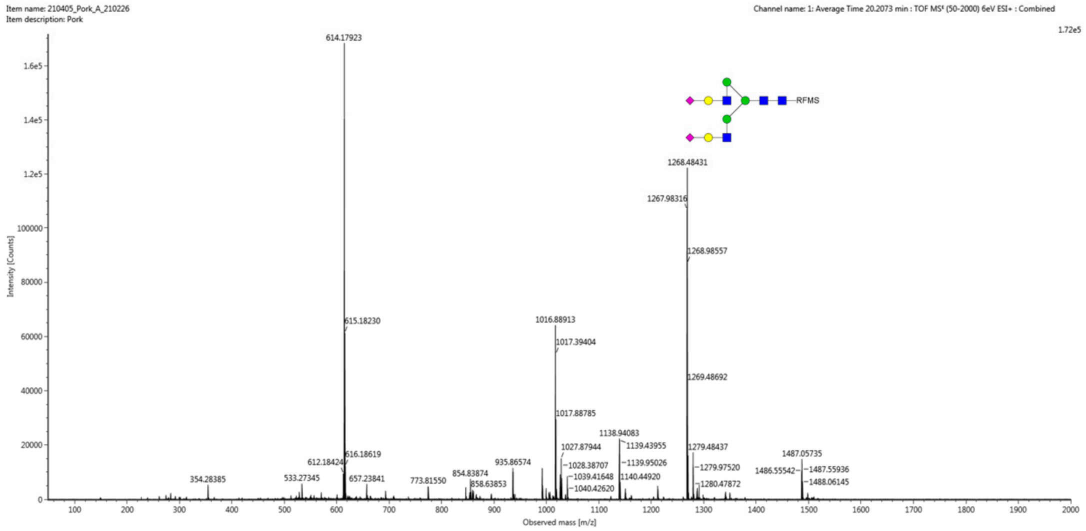

Figure S29. Pork: HexNAc(4)Hex(5)NeuAc(2) 2.3 ± 0.2%, 20.19 min.

Pork: HexNAc(2)Hex(8)
2.2±1.4% @ 20.48min

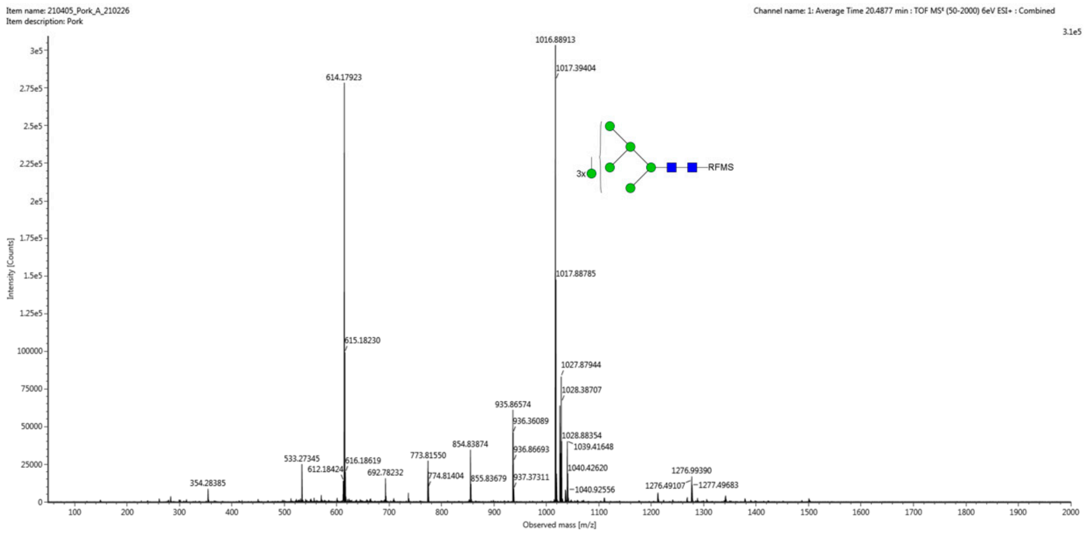

Figure S30. Pork: HexNAc(2)Hex(8) 2.2 ± 1.4%, 20.48 min.

Pork: HexNAc(4)Hex(7)NeuAc(1)
3.3±0.3% @ 20.8min

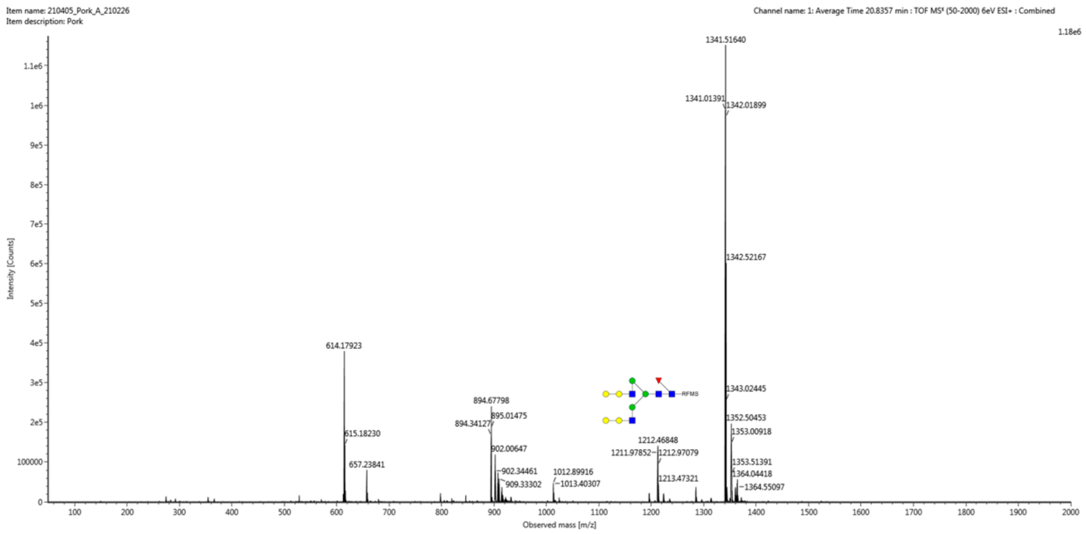

Figure S31. Pork: HexNAc(4)Hex(7)NeuAc(1) 3.3 ± 0.3%, 20.8 min.

Pork: HexNAc(4)Hex(6)Fuc(1)NeuAc(1)
60.7±6.3% @ 20.8min

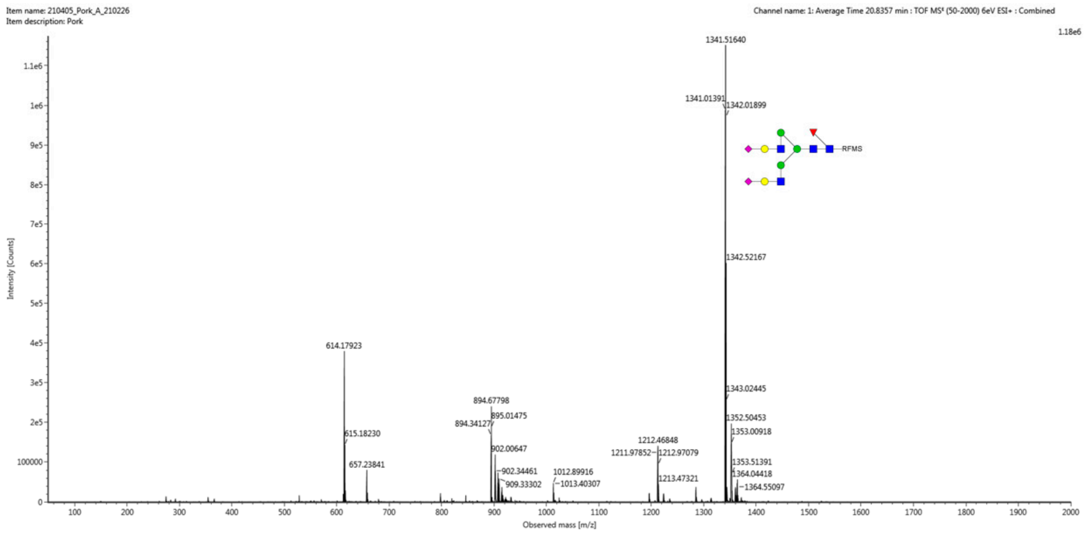

Figure S32. Pork: HexNAc(4)Hex(6)Fuc(1)NeuAc(1) 60.7 ± 6.3%, 20.8 min.

Pork: HexNAc(4)Hex(7)NeuAc(1)
1.1±0.1% @ 20.8min

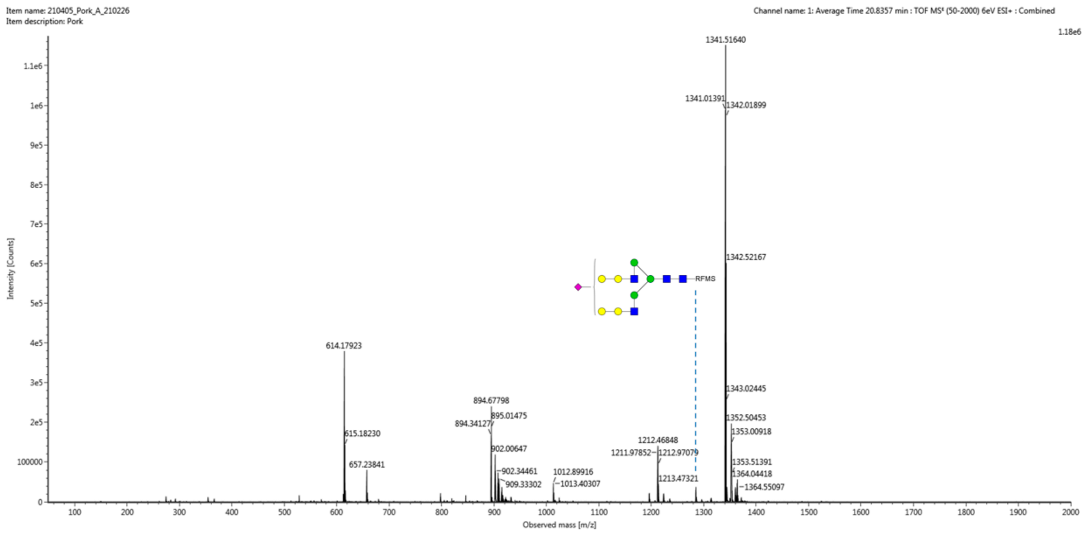

Figure S33. Pork: HexNAc(4)Hex(7)NeuAc(1) 1.1 ± 0.1%, 20.8 min.

Pork: HexNAc(4)Hex(5)Fuc(1)NeuAc(1)NeuGc(1)
4.9±0.3% @ 21.93min

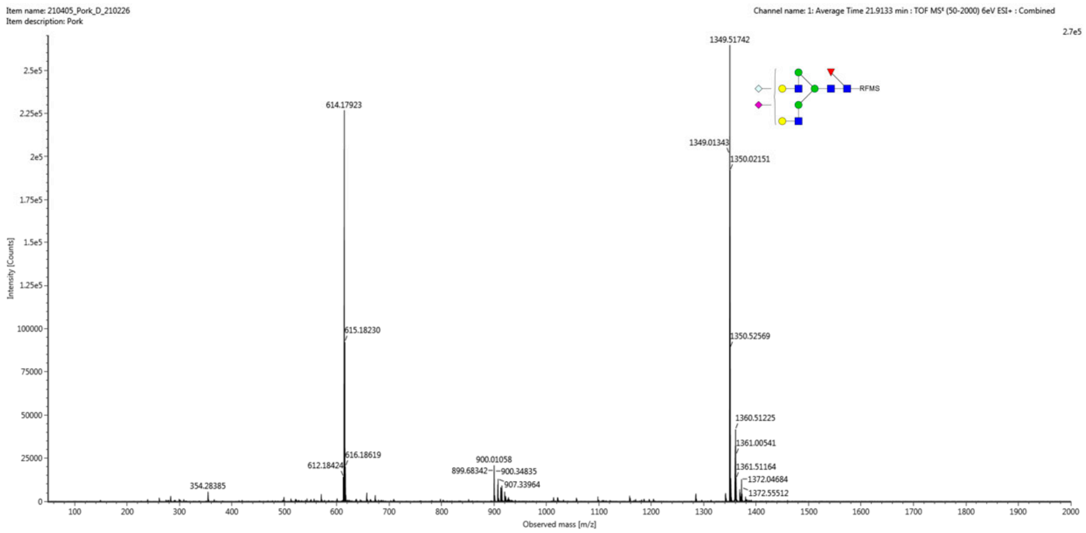

Figure S34. Pork: HexNAc(4)Hex(5)Fuc(1)NeuAc(1) 4.9 ± 0.3%, 21.93 min.

Pork: HexNAc(2)Hex(9)
2.3±1.4% @ 22.26min

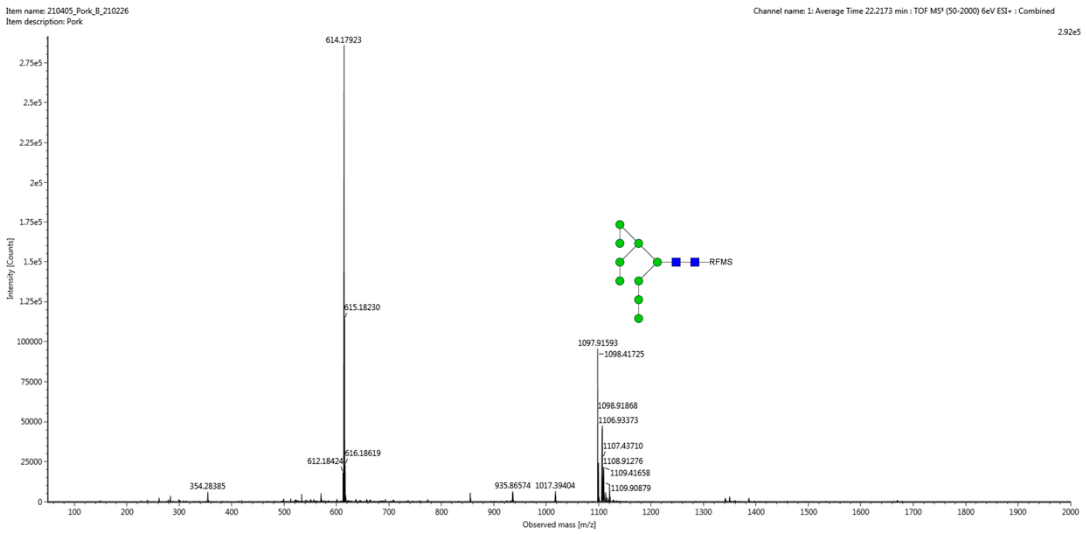

Figure S35. Pork: HexNAc(2)Hex(9) 2.3 ± 1.4%, 22.26 min.

Beef: HexNAc(2)Hex(4)
1.0±1.0% @ 9.89min

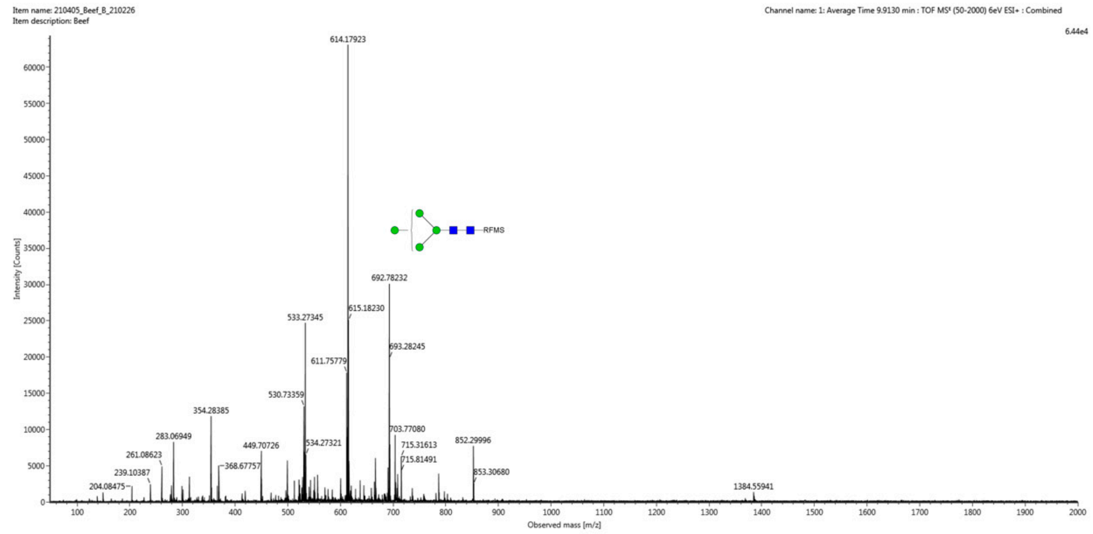

Figure S36. Beef: HexNAc(2)Hex(4) 1.0 ± 1.0%, 9.89 min.

Beef: HexNAc(4)Hex(3)Fuc(1)
0.8±0.5% @ 11.46min

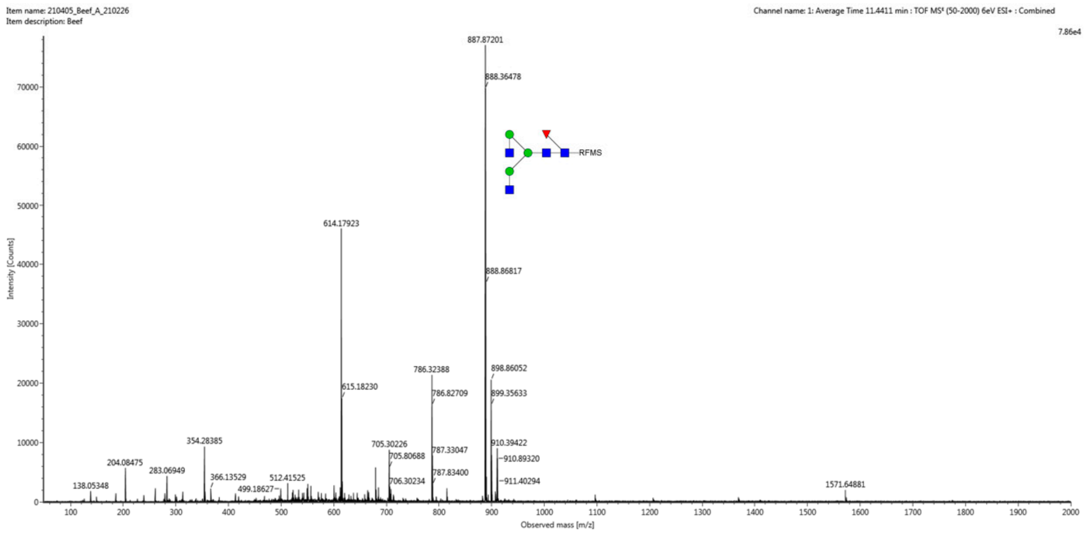

Figure S37. Beef: HexNAc(4)Hex(3)Fuc(1) 0.8 ± 0.5%, 11.46 min.

Beef: HexNAc(2)Hex(5)
5.8±0.3% @ 12.87min

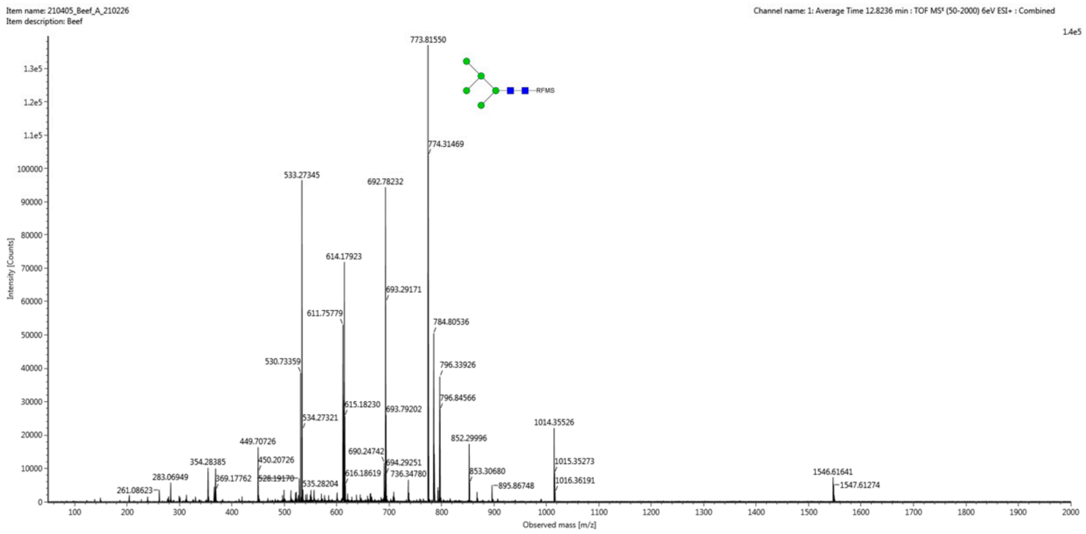

Figure S38. Beef: HexNAc(2)Hex(5) 5.8 ± 0.3%, 12.87 min.

Beef: HexNAc(4)Hex(4)Fuc(1)
1.8±1.4% @ 14.06min

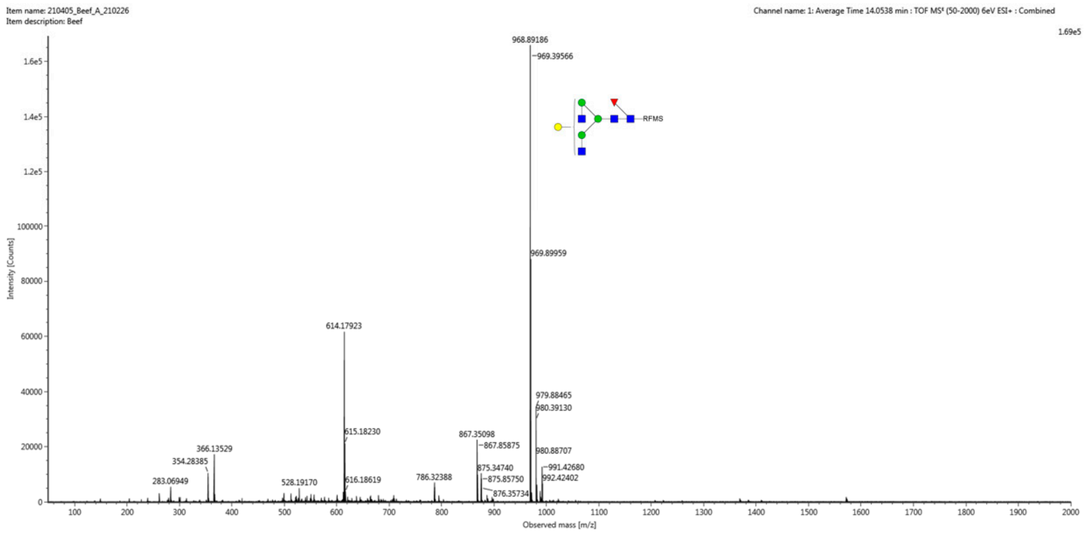

Figure S39. Beef: HexNAc(4)Hex(4)Fuc(1) 1.8 ± 1.4%, 14.06 min.

Beef: HexNAc(2)Hex(6)
4.1±1.4% @ 15.58min

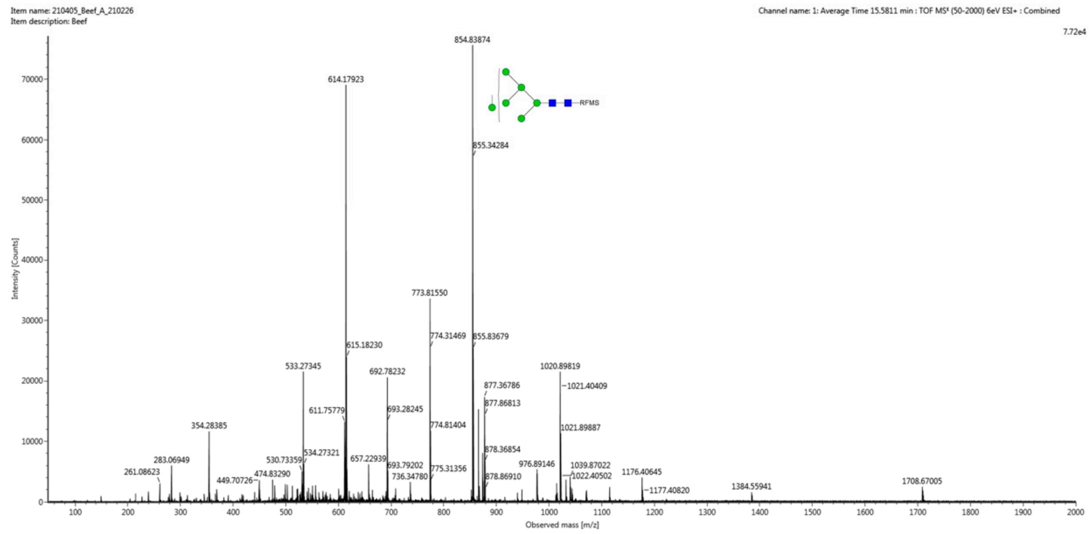

Figure S40. Beef: HexNAc(2)Hex(6) 4.1 ± 1.4%, 15.58 min.

Beef: HexNAc(4)Hex(5)Fuc(1)
2.5±0.5% @ 16.27min

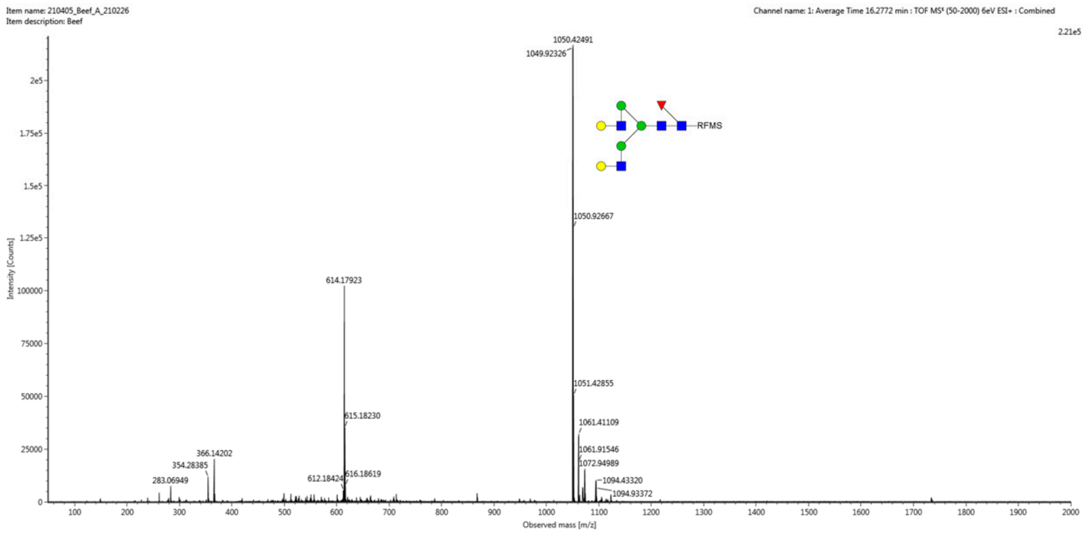

Figure S41. Beef: HexNAc(4)Hex(5)Fuc(1) 2.5 ± 0.5%, 16.27 min.

Beef: HexNAc(2)Hex(7)
4.1±0.9% @ 18.1min

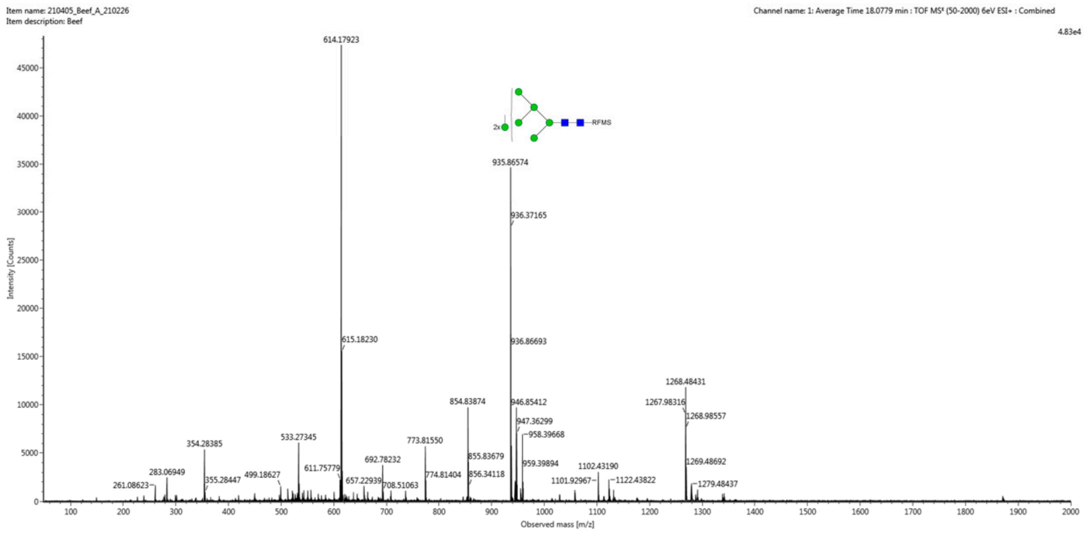

Figure S42. Beef: HexNAc(2)Hex(7) 4.1 ± 0.9%, 18.1 min.

Beef: HexNAc(4)Hex(5)Fuc(1)NeuAc(2)
4.6±0.1% @ 18.67min

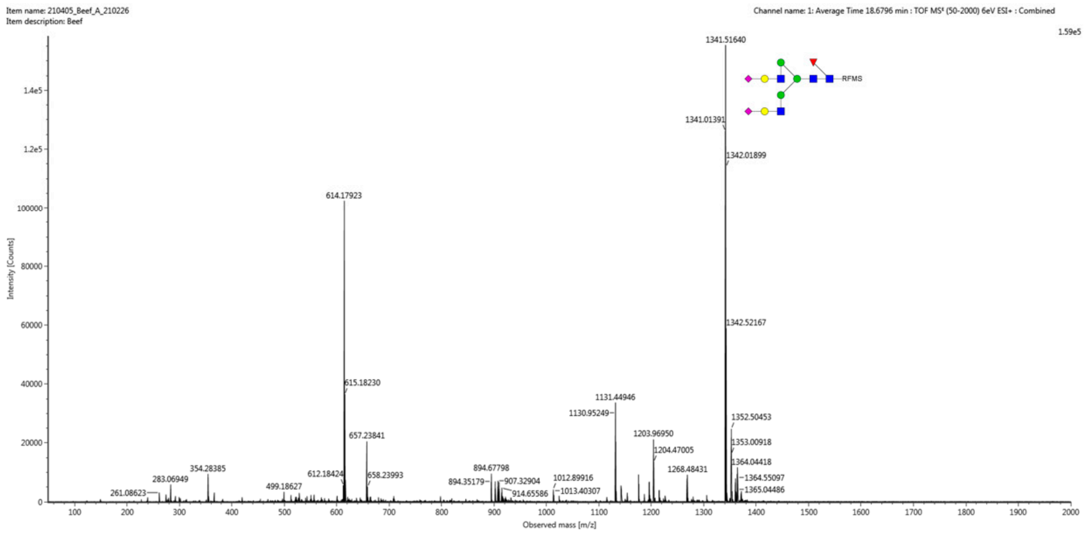

Figure S43. Beef: HexNAc(4)Hex(5)Fuc(1)NeuAc(2) 4.6 ± 0.1%, 18.67 min.

Beef: HexNAc(4)Hex(5)NeuAc(2)
9.7±1.0% @ 19.12min

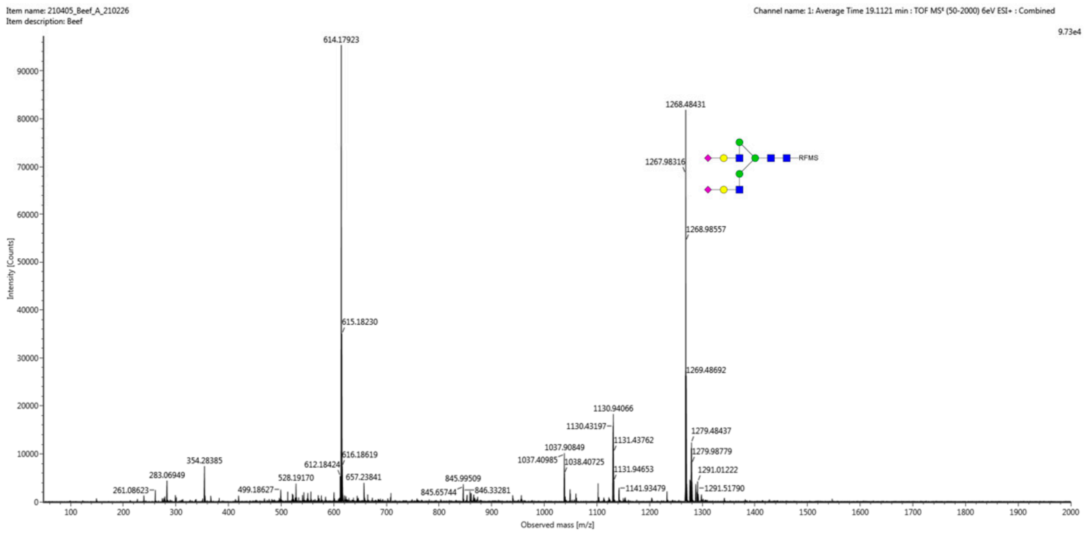

Figure S44. Beef: HexNAc(4)Hex(5)NeuAc(2) 9.7 ± 1.0%, 19.12 min.

Beef: HexNAc(4)Hex(6)Fuc(1)NeuAc(1)
8.1±0.7% @ 19.5min

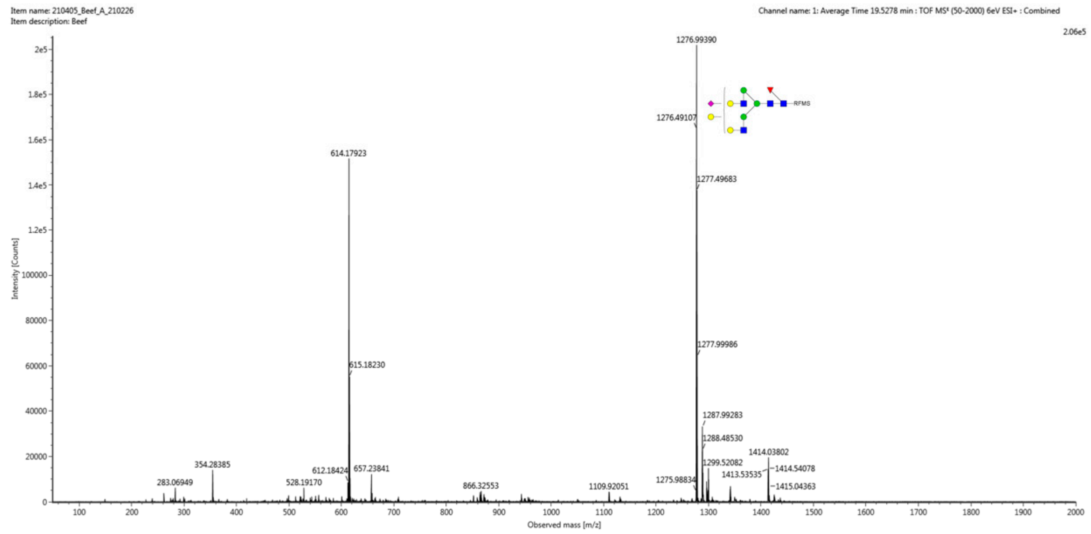

Figure S45. Beef: HexNAc(4)Hex(6)Fuc(1)NeuAc(1) 8.1 ± 0.7%, 19.5 min.

Beef: HexNAc(4)Hex(5)NeuAc(2)
9.7±1.0% @ 20.2min

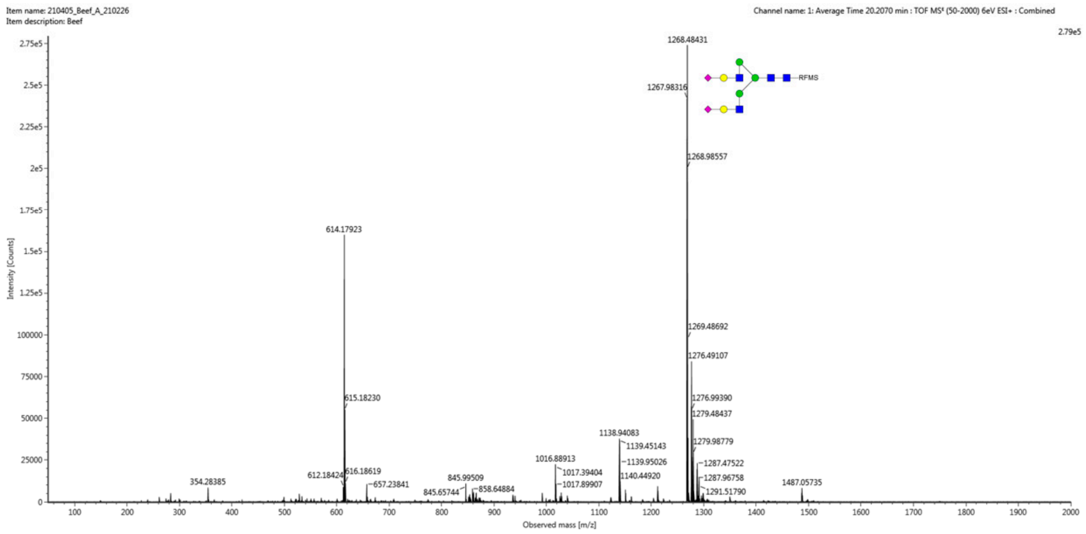

Figure S46. Beef: HexNAc(4)Hex(5)NeuAc(2) 9.7 ± 1.0%, 20.2 min.

Beef: HexNAc(2)Hex(8)
0.6±0.1% @ 20.2min

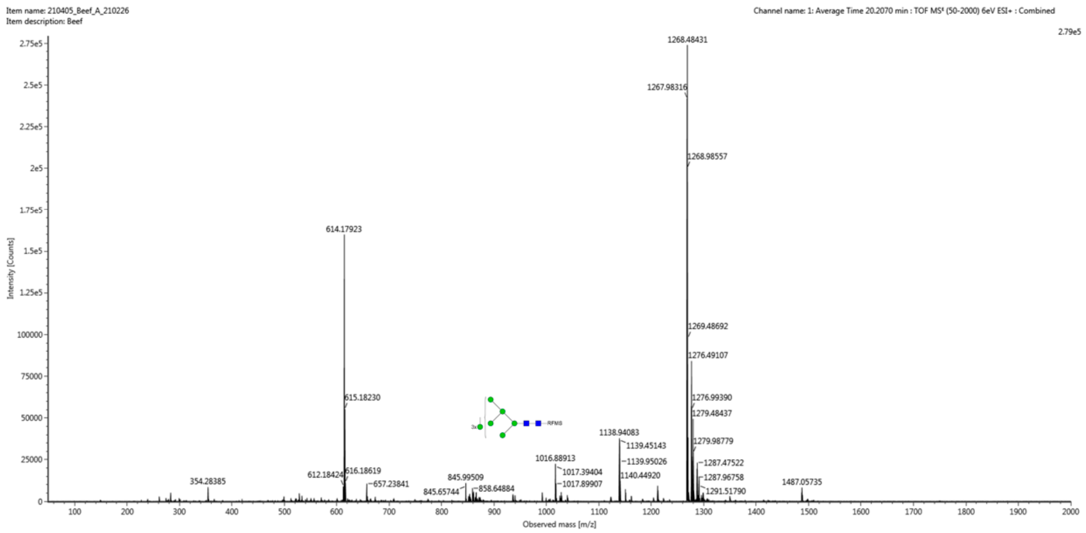

Figure S47. Beef: HexNAc(2)Hex(8) 0.6 ± 0.1%, 20.2 min.

Beef: HexNAc(4)Hex(7)Fuc(1)
17.8±1.3% @ 20.73min 20.82min

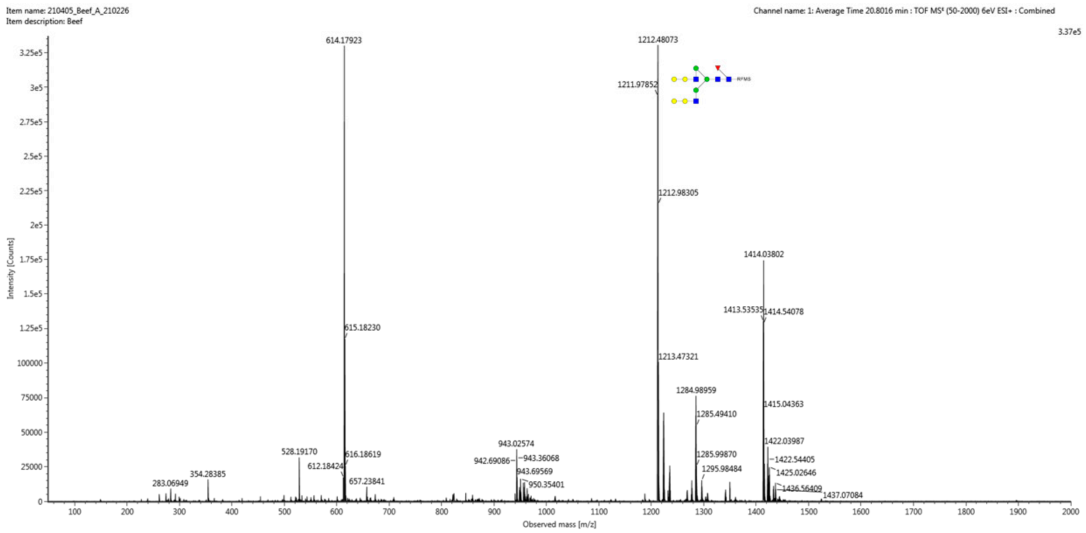

Figure S48. Beef: HexNAc(4)Hex(7)Fuc(1) 17.8 ± 1.3%, 20.73 min, 20.82 min.

Beef: HexNAc(4)Hex(5)NeuAc(1)NeuGc(1)
18.5±1.4% @ 21.53min

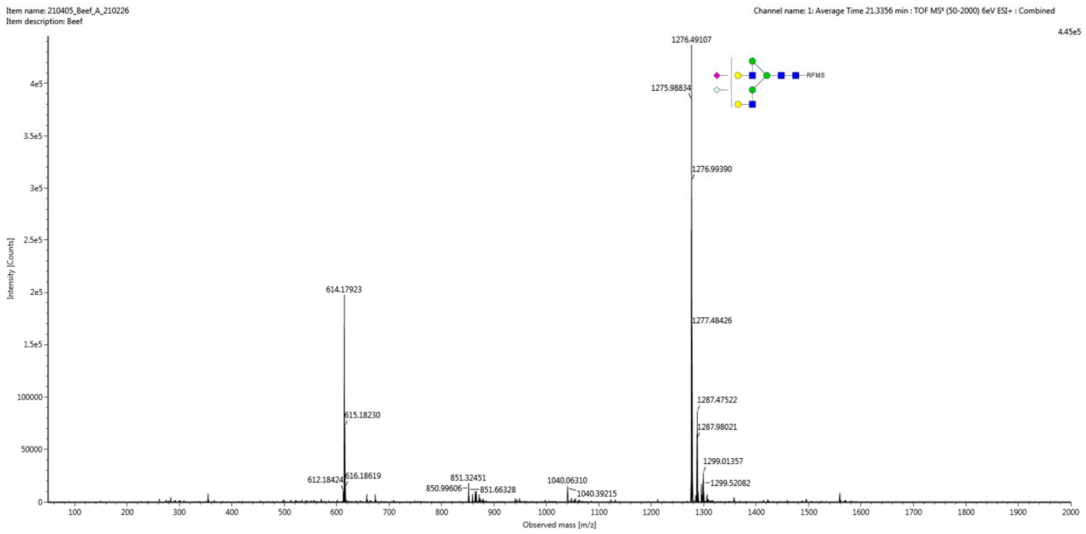

Figure S49. Beef: HexNAc(4)Hex(5)Fuc(1)NeuGC(1) 18.5 ± 1.4%, 21.53 min.

Beef: HexNAc(4)Hex(7)NeuAc(1)
2.3±0.2% @ 21.76min

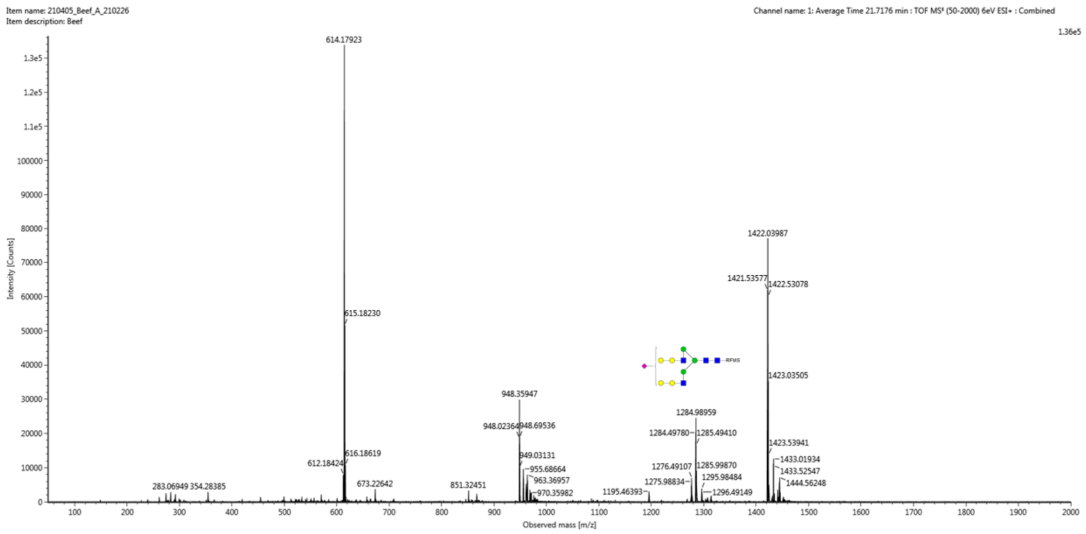

Figure S50. Beef: HexNAc(4)Hex(7)NeuAc(1) 2.3 ± 0.2%, 21.76 min.

Beef: HexNAc(4)Hex(6)Fuc(1)NeuGc(1)
4.2±0.4% @ 21.76min

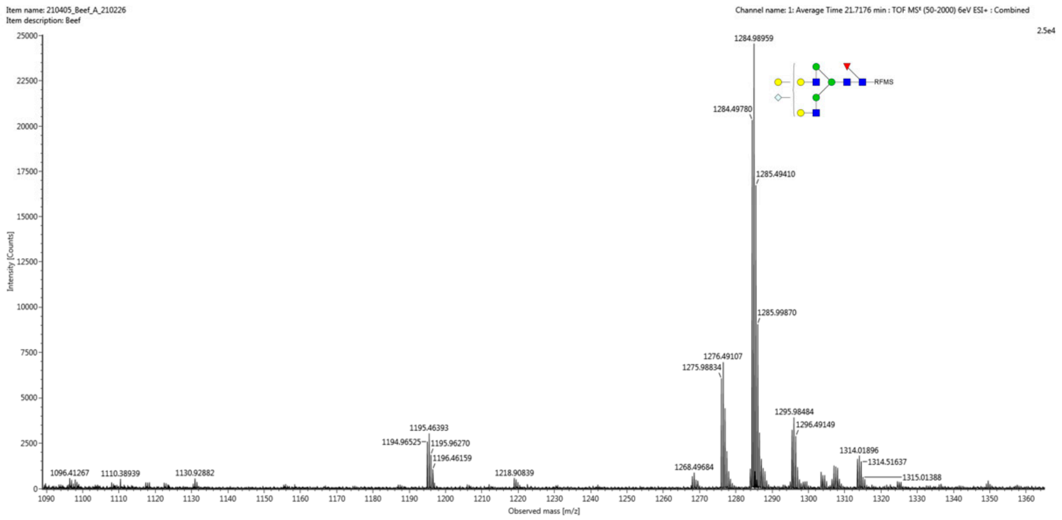

Figure S51. Beef: HexNAc(4)Hex(6)Fuc(1)NeuGC(1) 4.2 ± 0.4%, 21.76 min.

Beef: HexNAc(4)Hex(5)Fuc(1)NeuAc(1)NeuGc(1)
2.2±1.0% @ 21.86min

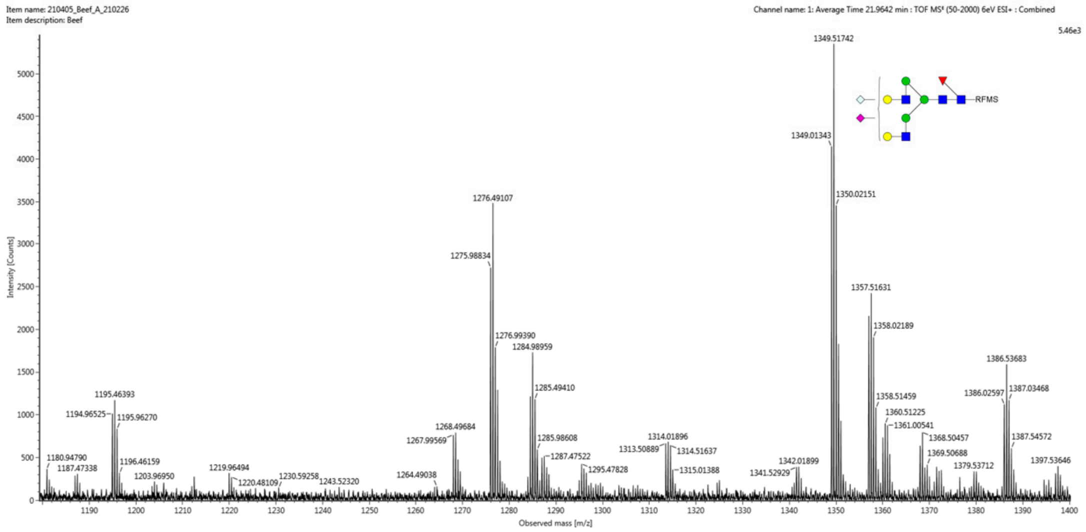

Figure S52. Beef: HexNAc(4)Hex(5)NeuAc(1)NeuGC(1) 2.2 ± 1.0%, 21.86 min.

Beef: HexNAc(4)Hex(5)NeuGc(2)
5.8±0.4% @ 22.5min

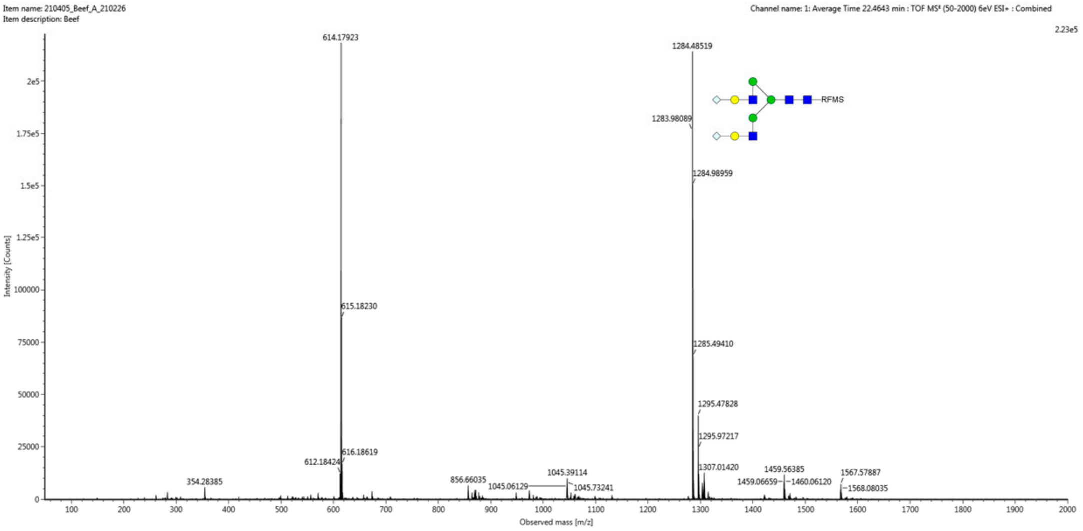

Figure S53. Beef: HexNAc(4)Hex(5)NeuGC(2) 5.8 ± 0.4%, 22.5 min.

Beef: HexNAc(4)Hex(5)Fuc(1)NeuGc(1)
2.5±1.0% @ 23min

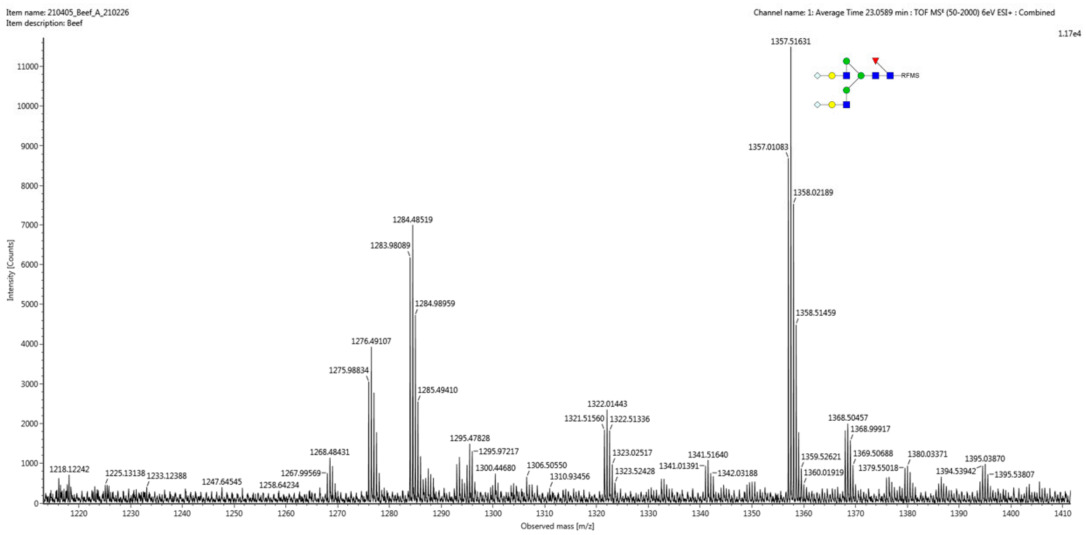

Figure S54. Beef: HexNAc(4)Hex(5)Fuc(1)NeuGC(1) 2.5 ± 1.0%, 23 min.
